# *C*‑methylated Flavanones with Antitrypanosomal Activity Isolated of Geopropolis from *Melipona mondury*

**DOI:** 10.1021/acsomega.5c01804

**Published:** 2025-05-27

**Authors:** Rafael Ferreira dos Santos, Afonso Santine M. M. Velez, Gabriel Fulgencio dos Santos, Paulo Pitasse-Santos, Bruno Sena de Oliveira, Marco Edilson Freire de Lima, Carlos Mauricio R. Sant’Anna, Raimundo Braz Filho, Debora Decote-Ricardo, Rosane Nora Castro

**Affiliations:** † Instituto de Química, Departamento de Química Orgânica, 67825 Universidade Federal Rural do Rio de Janeiro, CEP 23890-000 Seropédica, RJ, Brasil; ‡ Leicester Institute of Structural and Chemical Biology, University of Leicester, Leicester LE1 7RH, U.K.; § School of Chemistry, University of Leicester, Leicester LE1 7RH, U.K.; ∥ Instituto de Química, Departamento de Química Fundamental, Universidade Federal Rural do Rio de Janeiro, CEP 23890-000 Seropédica, RJ, Brasil; ⊥ Instituto de Veterinária, Universidade Federal Rural do Rio de Janeiro, CEP 23890-000 Seropédica, RJ, Brasil

## Abstract

Geopropolis is a natural antibiotic produced by stingless bees, supplemented with earth and clay. Bee products are a promising source of natural bioactive compounds with potential for the development of new drugs for neglected tropical diseases, such as Chagas disease. This study analyzed extracts of *Melipona mondury* geopropolis from the Bosque da Barra da Tijuca (RJ, Brazil) and evaluated their activity against *Trypanosoma cruzi*. The extracts were obtained using commercial grain alcohol 96° GL and fractionated with hexane and dichloromethane. The antichagasic activity was determined, with IC_50_ values ranging from 4.54 to 53.02 μg/mL. Phytochemical investigation of the dichloromethane fraction resulted in the isolation of two *C*-methylated flavanones, (2*S*)-strobopinin and (2*S*)-cryptostrobin, which were identified for the first time in Brazilian geopropolis. These compounds showed IC_50_ values of 21.21 μM and 21.55 μM, respectively, with distinct cytotoxities: 33.80 μM for strobopinin and 99.79 μM for cryptostrobin. Molecular modeling studies were conducted to propose a mechanism of action on a candidate enzyme target, glyceraldehyde-3-phosphate dehydrogenase, and possible reasons for the selectivity differences caused by *C*-methylation. The results highlight *M. mondury* geopropolis as a promising natural source of antiparasitic agents, supporting its potential for drug development.

## Graphical abstract

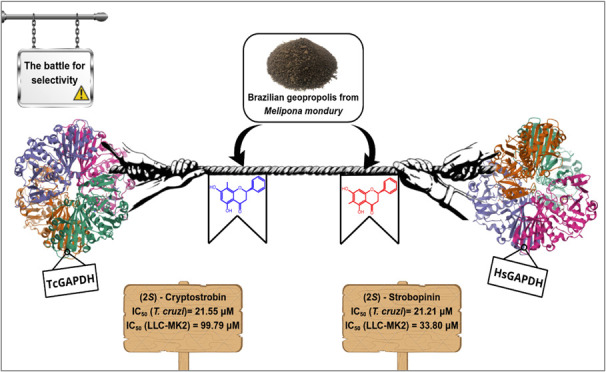

## Introduction

Natural products, especially bee products, have contributed significantly to the supply of bioactive substances used in treating various diseases that affect animals and humans.^1^ Among the products bees produce geopropolis stands out, which has increasingly motivated the scientific community to develop new studies to understand the chemical composition and potencial biological properties of this resin.^2^

Geopropolis, also known as batume, consists of a mixture of vegetable resins with clay, made by different species of stingless bees, designed to seal cracks, form the walls and entrance of colonies in hives, and mummify insects that come to invade it, preventing the occurrence of possible putrefaction due to the proliferation of fungi and bacteria.^3,4^

There are more than 400 species of stingless bees in Brazil, with the genera *Plebeia*, *Trigona*, *Melipona*, *Scaptotrigona*, and *Trigonisca* being the best-known. These bees play an essential role in preserving biodiversity, pollinating around 40 to 90% of floral species in Brazilian territory.^5,6^

Geopropolis has been used in traditional medicine for centuries in several countries, including Mexico, Brazil, Argentina, India, and Vietnam. This material is utilized in the treatment of various diseases due to its numerous therapeutic properties, including antioxidant, antimicrobial, anti-inflammatory, antiparasitic, gastroprotective, and immunomodulatory activities, which makes it a multifunctional matrix of interest for the isolation of substances with biological activities.^7,8^

The chemical composition of geopropolis is closely tied to the local flora. Furthermore, other factors also influence the chemical composition of this matrix, such as the season, climate, seasonality, soil composition, and the genetic characteristics of the bees themselves.^9^ Thus, geopropolis behaves as a matrix of high chemical complexity, which justifies the need for consistent data regarding the chemical substances responsible for the biological properties presented by this product, particularly those produced by the stingless bee species *Melipona mondury*, as there are few studies on this species of bee.^10,11^

Therefore, considering the presence of various species of stingless bees and numerous plant species in Brazilian territory, phytochemical investigations and biological analyses of geopropolis should be increasingly conducted so that the potential therapeutic use of this natural matrix can be used with complete safety.^9^ Using geopropolis as a promising source of bioactive natural substances presents an innovative alternative for the research and development of new drugs targeting various illnesses, with a particular emphasis, on neglected tropical diseases, such as Chagas disease (CD). Data available in the literature demonstrate the great potential of propolis’ antikinetoplastid activity, regardless of its geographic origin. These data stimulate different approaches to developing new drugs for treating infections caused by these protozoa. The most common human diseases caused by these parasites are Human African Trypanosomiasis, caused by *Trypanosoma brucei* subspecies *T. b. rhodesiense* and *T. b. gambiense*, American Trypanosomiasis (Chagas disease), caused by *Trypanosoma cruzi*, and the various forms of leishmaniasis caused by different species of *Leishmania*.^12^

CD, also known as American trypanosomiasis is endemic in Latin America and falls into the neglected tropical diseases listed by the World Health Organization (WHO), with approximately 6 to 7 million people infected worldwide. This infection is caused by the flagellated hemoprotozoan *Trypanosoma cruzi* (Kinetoplastida, Tripanosomatidae), which is transmitted to the human host, by the hematophagous vector known as “kissing bug” (*Triatoma infestans*, *Panstrongylus megistus*, among others).^13,14^

*T. cruzi* has a complex life cycle, with phases occurring both in the invertebrate vector and the vertebrate host. During the blood meal, the infected insect vector releases metacyclic trypomastigotes in its feces close to the bite site. Such evolutionary forms can be introduced into the host through the wound caused by the insect bite or the mucous membranes. In the host, parasites in the trypomastigote form infect cells close to the inoculation site, differentiating into amastigotes. Amastigotes are intracellular forms that replicate by binary fission. After multiplication, they differentiate into trypomastigotes and rupture the infected cells, being released into the bloodstream. Blood trypomastigotes can infect different cell types in different tissues, mainly muscle cells (cardiac, smooth, and skeletal) and ganglion cells, among others.^15^

The only drug commercially available for treating this parasitic disease in Brazil in its acute phase is benznidazole (Rochagan, Roche). However, in addition to its low effectiveness in chronic patients, with a cure rate of 10 to 20%, this drug has several side effects, such anorexia, nausea, vomiting, skin allergy, and peripheral neuropathy, which may even lead to treatment interruption.^16^

Therefore, given the need to investigate the chemical constituents of geopropolis, and also the limited and inefficient therapeutic arsenal available to treat chronic CD patients, this study aimed to evaluate the chemical composition of geopropolis produced by the bee species *Melipona mondury* (uruçu-amarela), and promote the isolation and structural elucidation of bioactive metabolites from the biomonitored fractionation of this extract, in addition to the evaluation of their activity against *T. cruzi* (Tulahuen C2C4-LacZ strain).

## Results and Discussion

### Antiparasitic Activity of the Crude Extract and its Fractions

The ethanolic extract of geopropolis (EEGP) was obtained by exhaustive extraction of the raw material from the geopropolis of *M. mondury*, yielding 17.2%. The EEGP was then partitioned using an eluotropic sequence of solvents to obtain its fractions, whose chemical profile was analyzed by HPLC (See Supporting Information). Additionally, the fractions were subjected to *in vitro* assays to evaluate their activity against *T. cruzi*, as described in the Experimental Section of this study.

The results of the *in vitro* tests carried out against amastigote forms of *T. cruzi*, the cytotoxic activity against LLC-MK2 cells, as well as the relationship between the cytotoxicity and antiparasitic activity (IS) of the geopropolis samples (EEGP and the HEX, DCM and MeOH: H_2_O fractions) are shown in Table 1. The extracts in this study were classified as highly active (IC_50_ < 10 μg/mL), active (10 μg/mL ≤ IC_50_ ≤ 50 μg/mL) and moderately active (50 μg/mL < IC_50_ ≤ 100 μg/mL), as described by Dutra et al. (2023).^17^ All the extracts showed activity against *T. cruzi* amastigotes, with IC_50_ values ranging from 4.54 to 53.02 μg/mL.

**Table 1:** Average IC_50_ Values Calculated for the Crude Extract and the Fractions Obtained

samples	*T. cruzi* (IC_50_ μg/mL)	cytotoxicity (IC_50_ μg/mL)	selectivity index (SI)
EEGP	4.79 ± 1.33	18.77 ± 1.44	3.92
Fr. HEX	4.54 ± 1.31	15.63 ± 1.21	3.44
Fr. DCM	10.45 ± 1.61	35.02 ± 3.45	3.35
Fr. MeOH:H_2_O	53.02 ± 7.00	106.20 ± 5.37	2.00
Benznidazole^a^	1.50 ± 0.33 μM	>200 μM	

a Reference drug. Selectivity = IC_50_ Cytotoxicity/IC_50_ *T. cruzi*. *n* = 3.

EEGP, the crude extract that gave rise to the fractions also evaluated in this study, showed promising antiparasitic activity, with an IC_50_ of 4.79 μg/mL. Fr-HEX proved to be the most active fraction from the liquid–liquid partitioning process, with an IC_50_ value of 4.54 μg/mL. However, there was no significant difference in IC_50_ compared to EEGP, so they were classified as highly active. Fr-DCM also exhibited antiparasitic activity against *T. cruzi* amastigotes, but less potent than Fr-HEX and EEGP, with an IC_50_ of 10.45 μg/mL, and was classified as active.

Fr-MeOH: H_2_O, which contains the most polar chemical components of the crude extract, was found to be moderately active, with an IC_50_ of over 50 μg/mL (53.02 μg/mL). This may be associated with the greater difficulty high-polarity chemical compounds have in crossing cell membranes, which justifies their lower trypanocidal activity compared to the other fractions evaluated in this study.^18^

The IC_50_ values obtained in this study demonstrate the encouraging antichagasic activity of geopropolis from *M. mondury*. These results are very relevant to the search for new natural bioactive compounds effective in the treatment of CD, especially as this is a pioneering study related to the investigation of the trypanocidal potential of a Brazilian geopropolis against this disease.

In comparison with the results obtained by Dutra et al. (2023) regarding the antiparasitic activity of extracts and fractions of red propolis from the Brazilian Amazon against promastigote forms of *Leishmania amazonensis*, the geopropolis from *M. mondury* proved to have better antiparasitic activity. The crude extracts of red propolis (EHPV-1, EHPV-2, EHPV-3 and EHPV-4) showed IC_50_ ranging from 23.37 to 36.10 μg/mL, which were higher than the IC_50_ presented by the ethanolic extract of geopropolis from *M. mondury* (EEGP) (4.79 μg/mL), and were classified as active, rather than highly active like EEGP.

In relation to the fractions obtained by the authors in the liquid–liquid partition process, the hexane fraction (FrHX) showed an IC_50_ of 45.99 μg/mL, whereas the hexane fraction of geopropolis from *M. mondury* exhibited 10 times better trypanocidal activity (4.54 μg/mL). The chloroform fraction (FrCL), which contains the medium-polarity chemical components of the red propolis extract, showed a slightly lower IC_50_ than the Fr-DCM in this study, remove at 16.11 μg/mL. The most polar fraction (FrEA) from the previous study had a higher IC_50_ value than Fr-MeOH: H_2_O in this study, and was classified as inactive because it had an IC_50_ above 100 μg/mL (112 μg/mL). However, both of the more polar fractions from these study (Fr-MeOH: H_2_O and FrEA) showed the lowest trypanocidal activity among the series of extracts evaluated.

Several samples of propolis from the state of São Paulo were evaluated for their activity against trypomastigote forms of *T. cruzi*. According to the authors, the sample extracted with absolute alcohol using the Soxhlet method had the highest concentration of bioactive compounds, resulting in the most significant activity, with an LD_50_ of 421.0 ± 26.5 μg/mL in 24 h.^19^

Silva et al. investigated three types of Brazilian propolis (red, green, and brown) against the Y strain of *T. cruzi* and found that all of them exhibited trypanocidal activity. However, only the red propolis maintained this activity after 96 h.^20^

Dutra et al. investigated the leishmanicidal activity of geopropolis from *Melipona fasciculata* collected from the Maranhão region. In this study, a significant reduction in infection of murine macrophages was observed, with a lower number of amastigotes internalized in cells treated with the geopropolis extract, compared to the untreated control group. Furthermore, the crude extract of this geopropolis was subjected to solvent partitioning in eluotropic order, with the ethyl acetate fraction showing the highest activity among those evaluated, also standing out for its higher selectivity index. Through gas chromatography coupled with mass spectrometry (GCMS), the phenolic compounds gallic and ellagic acids were identified as the main components in both the extract and the active fraction. These findings suggest that the antileishmanial activity of geopropolis is directly related to the presence of these compounds, emphasizing the relevance of phenolic acids in the biological properties of this matrix.^21^

The different biological activities observed in matrices of natural origin, such as bee products, are directly associated with the action of various classes of chemical substances. According to Sousa et al. (2019), the chemical composition of propolis is influenced by biotic and abiotic factors, such as seasonality, local flora, and the species of bee that produces it, which can significantly impact its bioactivity. In addition, variables such as extraction methods, type of solvent used, and extraction time also affect the amount of bioactive compounds present, influencing the therapeutic efficacy of the extract.^22^

It is worth noting that each geopropolis is unique, a singularity that arises from the vast number of native bee species capable of producing this resin, their wide distribution across the national territory, and Brazil’s rich floral biodiversity. In this context, Brazilian geopropolis stands out as an excellent alternative for the search for new bioactive substances with potential anti-Chagas activity.^9^

Thus, although the Fr-HEX fraction exhibited better antiparasitic activity compared to the other fractions, it also presented a more complex chromatographic profile, containing substances with very close retention times that were difficult to isolate. On the other hand, the Fr-DCM fraction, which also demonstrated notable antiparasitic activity, showed a simpler qualitative chemical profile by HPLC-PDA, with peaks in the chromatogram displaying UV curves with λ_max_ = 293 nm, resembling the structure of flavanones, which were easier to isolate.

Therefore, in our search to identify new natural antiparasitic agents in the geopropolis of *M. mondury*, we decided to preliminarily evaluate Fr-DCM, which exhibited better chromatographic resolution for the major components, as well as showing activity against *T. cruzi* and an IS comparable to Fr-HEX.

The Fr-DCM (328 mg) was subjected to VCL according to the Experimental Section described in of this paper. Of the subfractions collected, two groups appeared to be pure by TLC on silica gel, and after being dried in a rotary evaporator, they yielded whitish, needle-shaped solids weighing 15.0 mg (**S1**) and 21.3 mg (**S2**). The purity of substances **S1** and **S2** was determined by reverse-phase HPLC-PDA (>96%) and their structures were elucidated using one-dimensional (^1^H and ^13^C) and two-dimensional (HMBC) NMR and mass spectrometry (MS) techniques (See Supporting Information).

### Characterization of Flavanones

The main features observed in the NMR spectra of the isolated structures was a benzopyran-4-one skeleton, with saturation at positions 2 and 3 of the *C* ring, thus characterizing a typical flavanone structure, which was corroborated with data extracted by analysis of the UV curve in the HPLC-PDA (UV_max_ = 293 nm). Also observed was the presence of an unsubstituted *B* ring linked by a freely rotating bond to the *C* ring, which was confirmed by mass spectroscopy and the *A* ring containing two hydroxyl groups (5,7-dihydroxyl), one free and one chelated.

Our search for antiprotozoal clues led to the isolation of two flavonoids. By interpreting the spectral data obtained and comparing them with the literature, it was possible to identify **S1** as strobopinin (5,7-dihydroxy-6-methylflavanone) and **S2** as cryptostrobin (5,7-dihydroxy-8-methylflavanone). These are two *C*-methylated flavanones that have never been identified in Brazilian geopropolis, with a molar mass of 270 g/mol^23,24^ (Figure 1).

**Figure 1:**
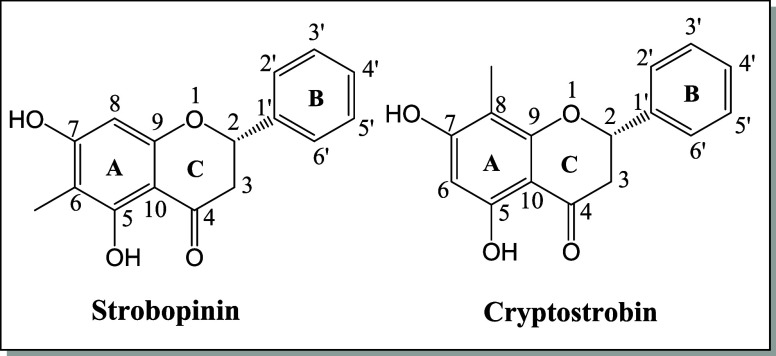
Proposed structures for substances **S1-** Strobopinin and **S2**- Cryptostrobin.

As these are chiral natural substances, optical rotation values were also obtained for the flavanones **S1** (2.4 mg/mL) and **S2** (1.5 mg/mL) in spectroscopic grade methanol. The values were −41.62° and −49.66°, respectively, indicating that they are levorotatory substances. By comparing these experimental results with those in the literature, it can be stated that the flavanones isolated in this work are (2*S*)-strobopinin and (2*S*)-cryptostrobin.^25^

Most of the signals present in the NMR spectra of the isolated substances were similar for both flavanones (**S1** and **S2**), such as the saturation of the *C* ring at the C-2 and C-3 positions, which presented a pair of diastereotopic hydrogens with double–doublet multiplicities: one at C-2 with δ 5.43 (*dd*, *J* = 12.0, 4.0 Hz) and 2H at C-3 with δ 2.84 (*dd*, *J* = 16.0, 12.0 Hz) and 3.11 (*dd*, *J* = 16.0, 4.0 Hz) for strobopinin (**S1**), and for cryptostrobin (**S2**) the H at C-2 with δ 5.40 (*dd*, *J* = 12. 0, 4.0 Hz), and another 2H at C-3 with δ 2.87 (*dd*, *J* = 16.0, 4.0 Hz) and 3.07 (*dd*, *J* = 16.0, 12.0 Hz). Both structures showed the hydroxyl of C-5 as a low-field singlet (12.30 and 12.0 ppm, respectively), due to intramolecular hydrogen bonding with the carbonyl in C-4, multiplets between 7.44 and 7.39 ppm, related to the hydrogens of the B ring, as well as a singlet at 2.1 ppm referring to its *C*-methylation.

For the ^13^C NMR spectrum, the chemical shifts were also similar for the two substances, differing only at the C-6 position for strobopinin (6.6 ppm) and at C-8 for cryptostrobin (7.4 ppm). Both showed a chemical shift at 196 ppm, characteristic of the carbonyl presents at the C-4 position, as well as the signals between 125–128 ppm relating to the carbons of the aromatic system (*B* ring). Signals were also observed between 160–163 ppm, which are related to the oxygenated aromatic carbons of the *A* ring (C9, C7, C5). The ^1^H-^13^C correlations in the HMBC were extremely important in distinguishing these two methyl-flavanones, which are position isomers, with the methyl group varying at the C-6 (**S1**) and C-8 (**S2**) positions of the *A* ring. The data indicated that the C-8 methyl group (δH 2.1) is correlated with the C-9 aromatic carbon (δC 159.9) for cryptostrobin. On the other hand, the two-dimensional technique showed that the methyl group at C-6 (δH 2.1) is correlated with the hydroxyl-containing carbon at C-5 (δC 162.6) for strobopinin. The chemical shifts are in agreement with previous reports described in the literature.^24^

In addition to the HPLC-PDA and NMR analyses, the *C*-methylated flavanones were also characterized by mass spectrometry (MS). The mass spectra of the natural flavanones exhibited intense peaks corresponding to the molecular ions: **S1** (*m*/*z* 270, [M+·]), **S2** (*m*/*z* 270, [M+·]). The nonsubstitution of the *B* rings in methylflavanones were confirmed by the presence of an ion corresponding to the phenyl cation with *m*/*z* 77 (6-Me 40%, and 8-Me 35%). The high-abundance ions with *m*/*z* 166 present in the compounds come from the Retro Diels–Alder (RDA) fragmentation mechanism, suggesting that *C*-methylation necessarily occurred in ring *A* (dihydroxybenzoyl) in positions 6 or 8, because positions 5 and 7 of this ring are substituted with two hydroxyl groups - one free and one chelated. The fragment ions with *m*/*z* 193 present in the mass spectra of the substances are characterized by the loss of the phenyl radical (ring *B*), helping to confirm that the methyl groups are present in positions 6 or 8 of ring *A* (**See** Supporting Information).

### Antiparasitic Activity of Isolated Flavanones

Studies indicate that propolis from both stingless and stinging bees possesses antiparasitic properties against various pathogenic protozoa, both intracellular and extracellular. In the study by Fonseca-Silva et al. (2011), the antileishmanial activity of quercetin, a flavonoid found in various propolis samples, was investigated against *Leishmania amazonensis* promastigotes. The results showed that quercetin inhibited parasite growth in a dose- and time-dependent manner, with an IC_50_ of 31.4 μM after 48 h of treatment.^26,27^

In another study by the same author, apigenin, a natural flavone also previously reported in propolis, was investigated for its antileishmanial activity *in vitro*, and its mechanism of action against *Leishmania amazonensis* promastigotes was described. Treatment with this flavone for 24 h resulted in dose-dependent inhibition of cell proliferation (IC_50_ = 23.7 μM) and increased generation of reactive oxygen species (ROS). According to the authors, apigenin also induced extensive mitochondrial swelling in the parasite, resulting in changes to the mitochondrial membrane potential, disruption of the trans-Golgi network, and cytoplasmic vacuolization. These findings demonstrate the leishmanicidal effect of apigenin and suggest that ROS-induced mitochondrial collapse is part of its mechanism of action.^28,29^

The literature also reports on the planning and synthesis of flavanones, evaluated against *T. cruzi* amastigotes. In work, a set of compounds with variations in substituents at different positions of the flavanone skeleton was prepared, enabling an interesting structure–activity relationship (SAR) study.^30^

In the study by Sousa et al., previously mentioned, the antichagasic potential of lyophilized red propolis was investigated. In the initial phase, the ethanolic extract of red propolis showed trypanocidal activity against *T. cruzi* epimastigote forms with an IC_50_ of 12.8 μg/mL.^31^ Similar results were observed by Dantas et al., highlighting the higher efficacy of red propolis extracts compared to other types. Subsequently, a similar procedure was conducted in this study with partitioning using different solvents.^32^ However, partitioning with chloroform resulted in higher activity (IC_50_ of 8.1 μg/mL) due to the higher content of flavonoids and aromatic acids, compounds present in higher concentrations in red propolis and associated with parasite inhibition. In the final stages, the PV–C2 and PV–C2–4 samples showed increased activity, with the compound vestitol presenting an IC_50_ of 5.2 μg/mL, belonging to the flavonoid class, known for its anti-*T. cruzi* properties.

The studies mentioned above emphasize that different classes of flavonoids have been evaluated as potential targets in an attempt to find new agents with anti-*T. cruzi* activity. Thus, in view of the social and economic impact caused by Chagas’ disease and the scarcity of effective chemotherapy drugs, especially in the chronic phase of the disease, the *C*-methylated flavanones isolated in this work, (2*S*)-strobopinin and (2*S*)-cryptostrobin, were also evaluated for their antiparasitic potential.

When tested against intracellular amastigote forms of *T. cruzi* (Table 2), a form of great clinical importance, both flavanones ((2*S*)-strobopinin and (2*S*)-cryptostrobin) showed significant trypanocidal activity, with IC_50_ values of 21.21 μM and 21.55 μM, respectively. However, the isolated substances showed very different cytotoxicity with IC_50_ values of 33.80 μM and 99.79 μM, respectively. These findings highlight the promising profile of (2*S*)-cryptostrobin for future antiparasitic treatments, revealed by its SI value (4.63).

**Table 2:** Average IC_50_ Values Calculated for the Isolated Methylflavanones

samples	*T. cruzi* (IC_50_ μM)	cytotoxicity (IC_50_ μM)	selectivity index (SI)
(2*S*)-strobopinin	21.21 ± 3.54	33.80 ± 1.52	1.59
(2*S*)-cryptostrobin	21.55 ± 1.88	99.79 ± 1.99	4.63
Benznidazole^a^	1.50 ± 0.33	>200	

a Reference drug. Selectivity = IC_50_ Cytotoxicity/IC_50_ *T. cruzi*. *n* = 3

Changing the position of the *C*-methyl group in the isolated flavanones did not result in a significant difference in antiparasitic activity, since both substances exhibited similar IC_50_ values. However, this modification was crucial in increasing the molecules cytotoxicity in LLC-MK2 host cells 3-fold. To investigate these experimental findings and explore a possible mechanism of action, computational studies were carried out, as described in the next section of this study, with a focus on molecular docking modeling.

### Molecular Modeling

Since there are many possible targets related to the flavanones activity against *T. cruzi*, determining the exact one is not an easy task, but literature results showed that TcGAPDH can be effectively inhibited by some flavonoids.^33^ An unequivocal proof of a proposed mechanism for the trypanocide activity of our compounds could be obtained, for example, by producing a recombinant protein target and measuring its inhibition by the compounds. Based on the literature results and on the structural similarity between our compounds and known TcGAPDH inhibitors,^33^ however, we considered the exploration of TcGAPDH as a candidate target by molecular modeling methods would be helpful in analyzing a possible mechanism of action for our compounds.

TcGAPDH catalyzes the sixth reaction of the glycolytic pathway of the trypomastigote and amastigote forms of *T. cruzi*, converting *D*-glyceraldehyde-3-phosphate into 1,3-bisphosphoglycerate. The enzyme has an orthosteric site for substrate binding, subdivided into two charge stabilization regions (Pi and Ps subsites). Adjacent to this site is the cofactor binding site, whose stabilization region for the NAD^+^ nicotinamide ring is a hydrophobic pocket, absent in the human enzyme, of about 10 Å in diameter, which contains amino acid residues exclusive for TcGAPDH, such as Ile13 and Tyr339. The Pi subsite has a serine residue in TcGAPDH, Ser247, which in the human enzyme is replaced by Ala232.

Initially, the cocrystallized ligand was redocked into the active site of TcGADPH including four different scenarios, with or without NAD+ and with catalytic residues in neutral or ionized forms. All GOLD fitness score functions were explored and the best results were obtained with the GoldScore function in the presence of NAD^+^ and with the catalytic residues in neutral form (RMSD = 0.947 Å). In the best redocking pose (Figure 2a), all main interactions described in the literature are conserved, including hydrogen bonds with residues Ser247, Thr226, Thr167, Thr199, Thr197 and Arg249.^34^

**Figure 2:**
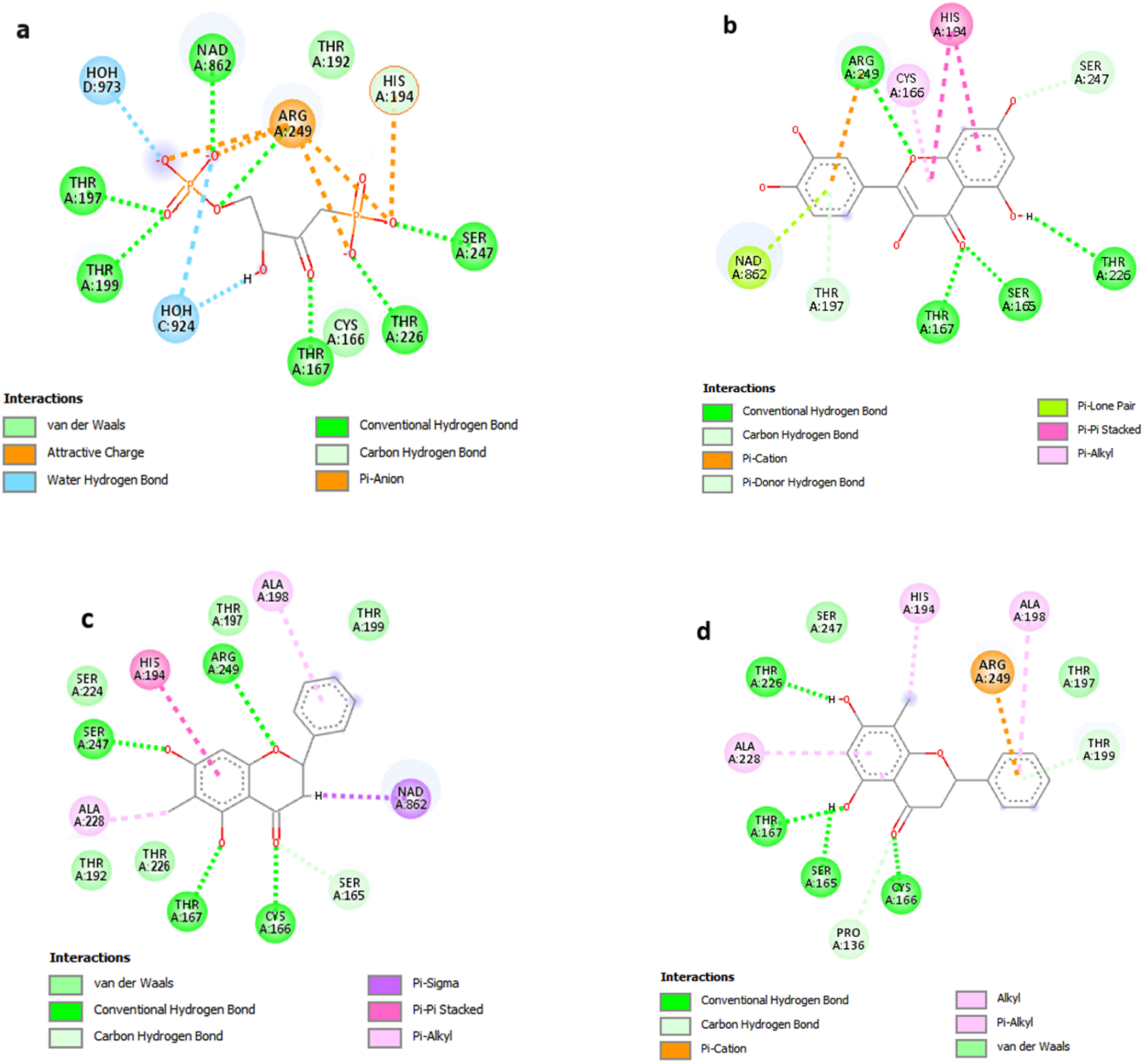
2D map of interactions in the active site of TcGAPDH for (a) reference GAPDH ligand, S70, after redocking with GoldScore; (b) quercetin best scoring pose (GoldScore); (c) strobopinin best scoring pose (GoldScore); (d) cryptostrobin best scoring pose (GoldScore). Figures generated with Discovery Studio, Dassault Systèmes, 2023.

Based on these results, the GoldScore function was chosen for the docking study in the presence of NAD+ and with the catalytic residues in neutral form. We also included as a reference in the molecular docking study quercetin, a flavonoid that holds substantial structural similarity with cryptostrobin and strobopinin, and which was shown to inhibit *T. cruzi* GAPDH (IC_50_ = 142 μM).^33^

The best poses obtained for the flavanones presented higher scores than the cocrystallized inhibitor, S70, and the flavonoid inhibitor, quercetin (Table 3), indicating that cryptostrobin and strobopinin are probably able to establish better interactions with TcGADPH than S70 and quercetin. Besides, part of the interactions that S70 carries out with the enzyme binding site are maintained in the docking poses obtained for quercetin, cryptostrobin and strobopinin (Figure 2).

**Table 3:** Best Docking Scores (Goldscore) for Ligands in TcGAPDH

ligand	fitness score (Goldscore)
reference (S70)	47.84
quercetin	50.14
cryptostrobin	54.06
strobopinin	55.75

To access the stability of the complexes with a more robust approach, the best docking poses had their geometries completely reoptimized at the semiempirical PM7 level to calculate ΔH_int_ values for the TcGAPDH-flavanone complexes, as described in the Methodology. The strobopinin and cryptostrobin complexes formation was enthalpically favorable in both cases, obtaining Δ*H*_int_ values of −307.76 and −317.60 kcal/mol, respectively (See Supporting Information). Although many factors may influence the trypanocide activity, at this point is interesting to compare the similarity of the Δ*H*_int_ values with the similarity of IC_50_ presented by both compounds (Table 2).

The interaction profiles of strobopinin (Figure 2b) and cryptostrobin (Figure 2c) in TcGAPDH contain significant differences as a result of the different position of the methyl group in their respective structures. The complete docking poses set of each compound indicated that the flavanones are able to interact with the orthosteric site, the cofactor site and the region between them, but the best scores were obtained when the ligands interact with the orthosteric site.

Strobopinin interacts with the enzyme through hydrogen bonds with Ser247, Thr167, Cys166 and Arg249, in addition to performing hydrophobic interactions with Thr226, Thr192, Thr197 and with the nicotinamide ring of the NAD^+^ cofactor (Figure 2b). There is also a π-stacking interaction with His194. The binding mode retained most of the interactions carried out by the reference inhibitor, but the interactions are stronger and there is also the π-stacking interaction, which is absent in the S70 complex and could be one of the reasons for the higher score of strobopinin. Another factor could be the higher flexibility of S70 in comparison with strobopinin, which could result in a more unfavorable fitness score because of the torsional potential included in GoldScore.^35^ The formation of a hydrogen bond with Thr167, combined with the interaction with the subsites Pi and Ps, were proposed as the main factors for satisfactory affinity and inhibitory activity.^34^

Cryptostrobin presented an interaction profile distinct from that of strobopinin, mainly due to the position of the *C*-methyl group that sterically prevents the interaction of the C ring ether group with the Arg249 guanidinium group, that is now involved in a cation-π interaction with the B ring. This steric hindrance reduced the interaction with the Pi subsite and caused a rotation of approximately 8° in ring *B*. New interactions with Pro136, Ser165, and a new H bond with Thr226 were formed (Figure 2c). Different from strobopinin, there is no interaction with the nicotinamide moiety of the NAD^+^. This situation could resemble the behavior of some 1,5-diphosphonopentanes without a substituent at the C-2 position in terms of a more restricted interaction with the Pi subsite, leading to a selective action against TcGAPDH.^34^

To test this hypothesis, strobopinin and cryptostrobin were also docked to the homologous human enzyme (HsGAPDH, PDB code: 1U8F; resolution = 1.75 Å) so as to evaluate the interaction mode and the interaction relationships with the Pi site (Figure 3). Strobopinin showed a good interaction with the Pi subsite of the human enzyme due to its interaction with Arg234 (residue homologous to Arg249 in TcGAPDH). Strobopinin presented several interactions with the active site, including with Ala232 (which is a serine residue, Ser247, of the TcGAPDH Pi subsite). However, cryptostrobin did not interact with Arg234, interacting minimally with the Pi subsite. This fact may be related with the observed selectivity of cryptostrobin against *T. cruzi* amastigotes in comparison with human LLC-MK2 fibroblasts.

**Figure 3:**
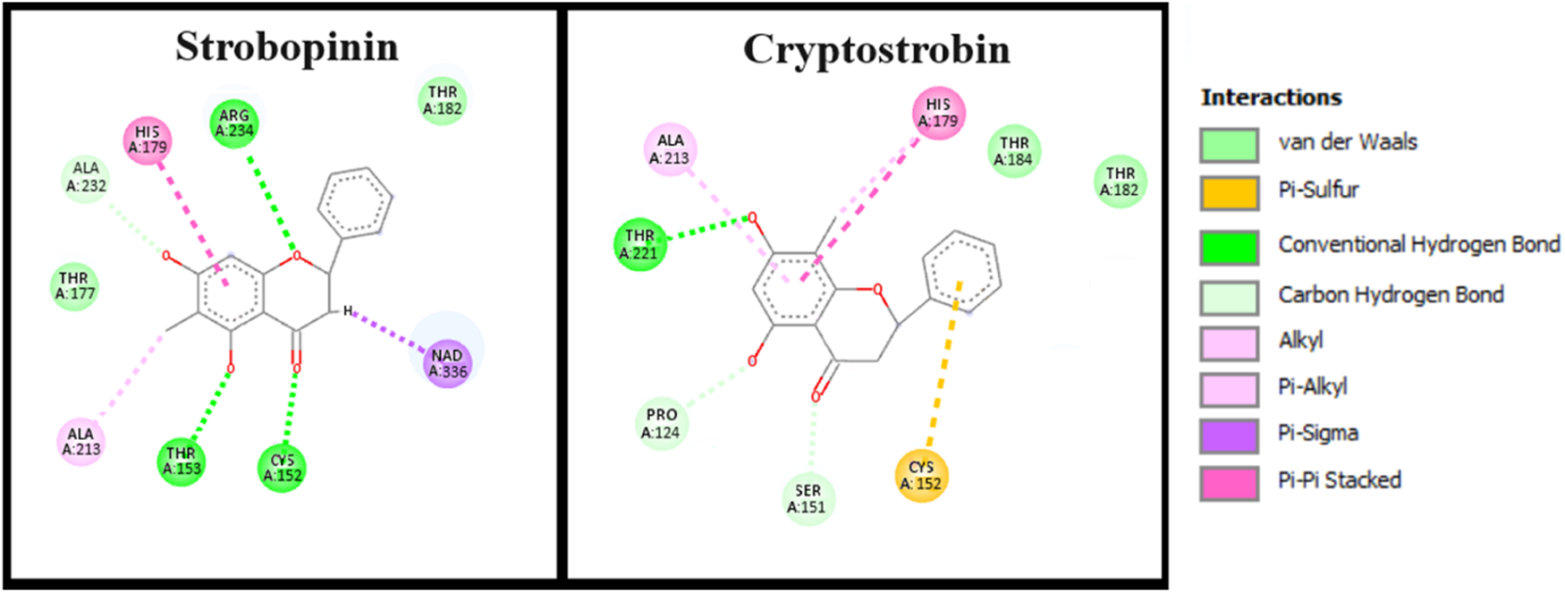
Best docking poses (GoldScore) of strobopinin and cryptostrobin, in the active site of HsGAPDH. Figures generated with Discovery Studio, Dassault Systèmes, 2023.

This could explain our experimental results that show that cryptostrobin, differently from strobopinin, is more toxic toward *T. cruzi* than human cells. For both flavanones, the docking pose is in accordance with the hypothesis of Yun and colleagues (2000) that the first site for inhibitor interaction is the Pi subsite.^36^ The docking results show that the most polar region of flavanones, those containing phenolic hydroxyls linked to ring A, interacts with the Pi subsite, and the most hydrophobic region (Ring *B*) extends toward the nicotinamide ring of the NAD^+^ cofactor, positioned in the enzyme hydrophobic pocket.

Finally, since the carbonyl carbon of strobopinin was located at a distance of 2.9 Å from the S atom of the catalytic Cys166, we decided to evaluate the possibility of a nucleophilic attack to form a covalent bond between ligand and enzyme. This possibility was verified through a covalent docking procedure with GOLD; since the addition would produce a chiral center at the *C* ring carbonyl carbon, both enantiomeric outputs were investigated. The ligand used as a scoring reference was *D*-glyceraldehyde-3-phosphate (GAP), the substrate of the GAPDH enzyme to which the Cys166 S atom makes a covalent bond during the enzymatic pathway. The results (Table 4) are suggestive of an enantiomeric preference for the attack that results in the (2*R*) product (Figure 4a). This complex presented a covalent bond distance of 1.85 Å, between C4 of the *C* ring and the Cys166 S. On the other hand, the (2*S*) enantiomer is destabilized by steric effects inside the orthosteric site. The same study was carried out for cryptostrobin, whose carbonyl carbon was located at a distance of 3.43 Å from the S atom of the catalytic Cys166 in the noncovalent complex, and the results are also consistent with an enantiomeric preference for the attack that results in the product (*R*). This complex presented a covalent bond distance of 1.84 Å, between C4 of the *C* ring and the S of Cys166 (Figure 4b).

**Figure 4:**
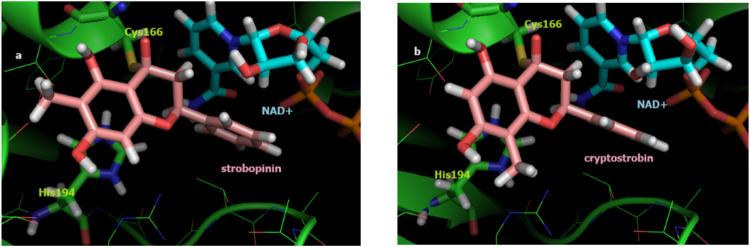
(a) Best covalent docking pose of strobopinin (sticks, C atoms in pink) into *T. cruzi* GAPDH (cartoon); (b) best covalent docking pose of cryptostrobin (sticks, C atoms in pink) into *T. cruzi* GAPDH (cartoon) (catalytic residues Cys166 and His194, sticks, C atoms in green; NAD+, sticks, C atoms in cyan). Figure generated with PyMOL 0.99rc6 (DeLano Scientific LLC).

**Table 4:** Best scores (Goldscore) for covalent docking of ligands in TcGAPDH

ligand	fitness score (Goldscore)
*D*-glyceraldehyde-3-phosphate	80.02
strobopinin (2*R* enantiomer)	72.89
strobopinin (2*S* enantiomer)	41.47
cryptostrobin (2*R* enantiomer)	78.69
cryptostrobin (2*S* enantiomer)	59.63

### Prediction of ADME Properties

According to data from the theoretical pharmacokinetic study (Figure 5) with the swissADME server, strobopinin and cryptostrobin fit completely to Lipinski’s Rule of Five. They were predicted to have a good solubility and gastrointestinal absorption profile, in addition of showing the ability to cross the blood-brain barrier to act on the central nervous system. They do not display any warning about the formation of toxic metabolites after the pharmacokinetic phase of biotransformation, according to the Brenk parameter of Medicinal Chemistry. These flavanones can potentially inhibit the hepatic microsomal system, to a greater or lesser extent, acting on the enzymes CYP1A2, CYP2C19, CYP2D6 and CYP3A4, and their effects are not necessarily harmful, depending on the metabolic pathway in which the microsomal system is located.

**Figure 5:**
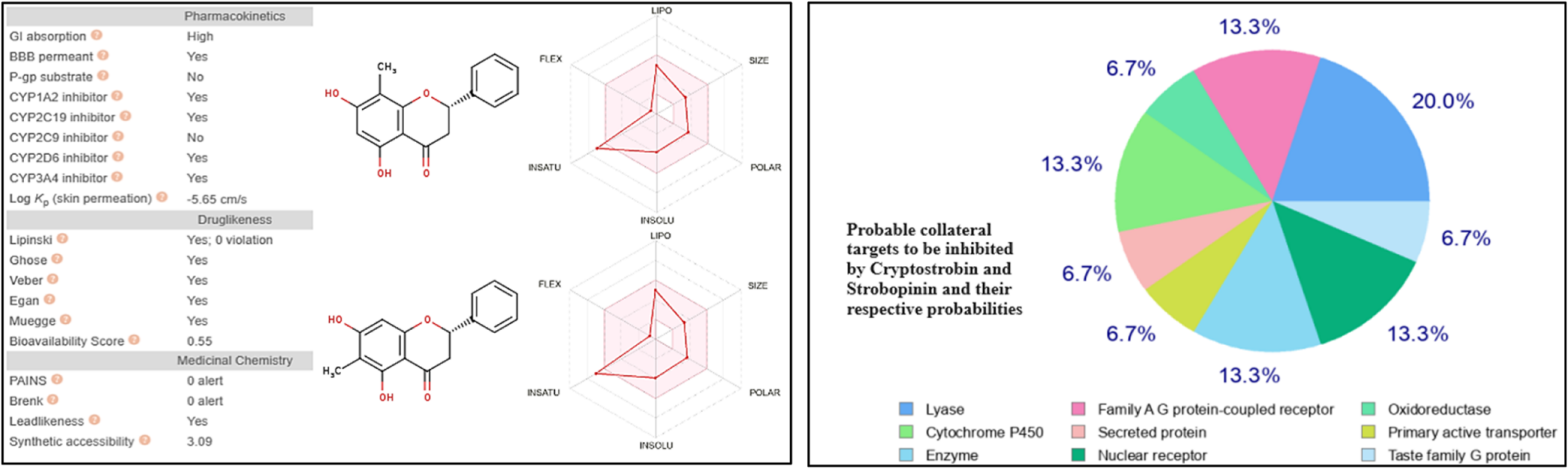
Predicted pharmacokinetic profile of strobopinin and cryptostrobin. Physicochemical parameters and delineation of the probable scope of inhibition of human metabolic enzymes obtained with Swiss ADME.

Therefore, it can be concluded that for a chemically complex matrix such as geopropolis, the use of semipurified extracts associated with VLC enabled the isolation of two natural methylflavanones from the dichloromethane fraction of the ethanolic extract of geopropolis from *Melipona mondury*, which showed encouraging antiparasitic activity. It is also worth highlighting the novelty of these substances in the work matrix, since these *C*-methylated flavanones are being reported for the first time in a Brazilian geopropolis. It is important to highlight the use of VLC, which is a simple, efficient and low-cost technique applied to the isolation of bioactive compounds in natural products. In this work, we employed molecular modeling tools to propose a possible mechanism of action of strobopinin and cryptostrobin that were shown to be active against *T. cruzi*, using TcGAPDH as a candidate target for their action, based on previous results in the literature.

Both compounds were predicted to interact favorably with TcGAPDH by molecular docking and semiempirical calculations, with quite similar fitness scores and interaction enthalpy values for both structures, in qualitative agreement with our experimental data that showed them to be equipotent against the parasite. The position of the methyl group at the *C*-ring in both flavanones was crucial for the respective interaction modes with TcGAPDH. We propose that the methyl group position in cryptostrobin impairs its interaction with the Pi subsite in human GAPDH, as a possible explanation of the selectivity against the parasite enzyme observed experimentally.

The application of molecular modification strategies to flavanones can be an important factor in reducing the interaction with the Pi subsite and ensuring greater selectivity, in addition to promoting the derivatization of the *B* ring, with the introduction of polar groups, to explore the interaction with the Ps subsite and fully optimize the affinity of these compounds on TcGAPDH. The mode of interaction of cryptostrobin highlighted the interaction with other amino acid residues seen as important new microtargets within the active site of the enzyme, which may be useful for designing new selective inhibitors of TcGAPDH.

## Conclusions

This study analyzed ethanolic extracts of Brazilian geopropolis from *Melipona mondury* and evaluated its activity against *Trypanosoma cruzi*. The extract was partitioned with hexane and dichloromethane, indicating the presence of active compounds. Two C-methylated flavanones, (2S)-strobopinin and (2S)-cryptostrobin, were isolated from the dichloromethane fraction and identified as novel components of this Brazilian geopropolis. Although both flavanones showed comparable levels of activity, (2S)-cryptostrobin proved to be the most selective compound. Molecular docking with experimental studies contributed to the elucidation of the mechanisms of action and selectivity of these natural products. This study reinforces the potential of geopropolis as a rich source of natural bioactives with important antiparasitic activity.

## Experimental Section

### General Experimental Procedures

The solvents used in this study were purchased from Sigma-Aldrich, Anidrol, Neon, Vetec or Merck. Silica gel 60 F-254 sheets with a thickness of 0.2 mm were used for the preliminary analyses using analytical thin layer chromatography (TLC). The chromatographic profile of the extracts and the substances isolated in this study was carried out on a Prominence liquid chromatograph (Shimadzu) with LC-20AT series pumps, SPD-M20A detector and SIL-10A autoinjector. The equipment was controlled and data acquired using LCSolution software (Shimadzu). The gas chromatography coupled to mass spectrometry (GCMS) analysis was performed using a Shimadzu QP2010 Plus mass spectrometer, which was connected to a gas chromatograph (model GC-17A). The analyses were carried out on a HP-5 fused silica capillary column (5% diphenyl-95% dimethylpolysiloxane, 30 m × 0.25 mm internal diameter × 0.25 μm film thickness, Agilent), in 30:1 split mode, with a transfer line at 290 °C, helium carrier gas at a flow rate of 1 mL·min^–1^. The mass spectrometer was operated with an electron impact detector with an energy of 70 eV, an interface temperature of 310 °C, an ionization source temperature of 250 °C, an oven temperature of 150 °C held for 1 min, followed by a heating rate of 10 °C per minute up to 290 °C, held for 20 min. The mass fragments were detected in a scan ranging from 40 to 600 in scan mode. For vacuum liquid chromatography (VLC), silica gel 60 was used for TLC with 13% gypsum, with a fluorescence indicator, and a sintered glass funnel 15 cm high and 4 cm in diameter. The optical rotations were measured on a Jasper P2000 polarimeter with a 10 dm cuvette, at room temperature. The compounds were prepared in a spectroscopic-grade methanol solution, and the optical rotation values obtained were compared with the literature. The Hydrogen Nuclear Magnetic Resonance (^1^H NMR) and Thirteen Carbon Nuclear Magnetic Resonance (^13^C NMR) spectra were obtained on a Bruker Ultrashield Plus spectrometer operating at 11.75 T (500 MHz for the ^1^H nuclei and 125 MHz for the ^13^C nuclei) and processed using Mestrenova (Version: 14.2.0-26256). The samples were solubilized in deuterated chloroform (CDCl_3_), with tetramethylsilane (TMS) as the internal reference standard. The chemical shifts were expressed in parts per million (ppm) (δ) and the coupling constants (*J*) were recorded in Hertz (Hz). The theoretical explanation for the trypanocidal activity and cytotoxicity displayed by the substances isolated in this study was obtained through computational methods, based on the docking process using the GOLD 2023.2.0 program (CCDC), at the orthosteric site of the enzyme glyceraldehyde-3-phosphate dehydrogenase (GAPDH). All the NMR spectra and chromatograms (GCMS and HPLC-PDA) of the isolated substances and extracts obtained in this study are provided in our Supporting Information.

### Geopropolis Samples

The samples of *in natura* geopropolis from *Melipona mondury* ( uruçu-amarela) were collected from a meliponary located in Bosque da Barra da Tijuca, Rio de Janeiro (23°25′S, 42°56′W). These samples were frozen, pulverized using a semi-industrial blender until a homogeneous, finely divided powder was obtained, and then stored in sealed bottles in the freezer until analysis.

### Preparation of Geopropolis Extract

The pulverized geopropolis sample (20 g) was extracted three times for 2 h each with 150 mL of commercial grain alcohol (96° GL) in an ultrasonic bath (40 kHz). The extract was then filtered to remove the inorganic fraction (soil). The three alcoholic extracts obtained were combined and concentrated using a rotary evaporator under reduced pressure at 45 °C until a constant weight was achieved, yielding the ethanolic extract of geopropolis (EEGP) (*m* = 3.44 g) used in the subsequent fractionation, chemical analysis and antiparasitic activity steps.

### Fractionation and Isolation of flavanones

The EEGP (950 mg) was solubilized in methanol: water (7:3 v/v) using an ultrasonic bath (UltraCleaner 1600; frequency: 40 kHz), at T.A. The partitions were carried out with 300 mL (5 × 60 mL) of hexane and dichloromethane, respectively. The fractions obtained were dried with anhydrous sodium sulfate (Na_2_SO_4_), filtered, and concentrated under reduced pressure to obtain Fr-HEX (580.4 mg), Fr-DCM (333.6 mg), and Fr-MeOH: H_2_O (17.5 mg).

All the fractions had their chemical profiles monitored by chromatographic analysis methods (HPLC-PDA and TLC) and were evaluated against *T. cruzi* strains to determine their antiparasitic potential.

Flavanones were isolated using vacuum liquid chromatography (VLC). A sintered glass funnel attached to a vacuum line was packed with TLC grade silica gel (40 g). The silica gel was compressed under vacuum to achieve a uniform layer, facilitating better separation. The sample was eluted as a slurry, using the mobile phases hexane, hexane: chloroform (1:1 v/v), chloroform, and chloroform: acetone (1%), respectively. Each fraction was collected in an Erlenmeyer flask. After the separation procedure, the obtained fractions were monitored by TLC, visualized under ultraviolet light at 254 nm, and developed using the destructive chemical reagent vanillin-sulfuric acid. Fractions with similar TLC profiles were pooled. The combined fraction was concentrated using a rotary vacuum evaporator.

## Biological Assays

Mammalian cells were used to evaluate the cytotoxicity of the tested compounds. LLC-MK2 cells (ATCC) were used and cultured in Dulbecco’s modified Eagle’s medium (DMEM) supplemented with 5% fetal bovine serum (FBS), being incubated at 37 °C (5% CO_2_), with successive passages every 4–5 days. Cells were dissociated from the monolayer by treatment with a solution containing 0.25% w/v trypsin and 0.04% EDTA.

The antiparasitic activity assays used the Tulahuen C2C4-LacZ strain of the *T. cruzi* parasite, being evaluated in the amastigote forms. Parasites of the morphologic form amastigotes and trypomastigotes were cultivated by successive reinfections in a monolayer of LLC-MK2 cells in DMEM medium +2% FBS, being incubated at 37 °C (5% CO_2_). Trypomastigote forms were collected from the culture supernatant between the fifth and 10th day after infection and separated from nonadherent cells by differential centrifugation.^37^

### Evaluation of Cytotoxicity against LLC-MK2 Cells

In a 96-well transparent plate, a suspension of 1 × 10^4^ LLC-MK2 cells (ATCC) in DMEM medium +2% FBS was added. Cells were incubated at 37 °C (5% CO_2_) for 20 h and then washed with PBS to remove nonadherent cells. Cells were treated with serial dilutions of the compounds in triplicates, prediluted in DMEM + 2% FBS. Untreated controls, vehicle (0.2% v/v DMSO), and blank (no added cells) were included in the experiment. After incubation for 120 h, the supernatant was removed, and the cell monolayer was washed with PBS, then the culture medium was renewed. Twenty μL of 3.0 mM MTT saline were then added, followed by incubation for another 1.5 h. The supernatant was then removed and the MTT formazan crystals were dissolved by adding 120 μL/well of DMSO. After incubation for 1.5 h to dissolve the MTT crystals away from light and at 37 °C, the absorbance was measured at λ = 570 nm using a plate reader.^37^

### Evaluation of Trypanocidal Activity Against Amastigote forms of *T. cruzi*

In a 96-well transparent plate, a suspension of 1 × 10^4^ LLC-MK2 cells (ATCC) in DMEM medium +2% FBS was added. Cells were incubated at 37 °C (5% CO_2_) for adhesion for 3 h and then washed with PBS to remove nonadherent cells. A suspension containing 1.5 × 10^5^ trypomastigotes of the *T. cruzi* Tulahuen C2C4 LacZ strain was added to the cells, followed by incubation at 37 °C (5% CO_2_) for 20 h to establish infection. Noninternalized parasites were removed by three successive washes with PBS, followed by treatment with serial dilutions of the compounds in triplicates, prediluted in DMEM + 2% FBS. Untreated, vehicle (0.2% v/v DMSO) and blank (no added parasites) controls were included in the experiment. Benznidazole was used, in serial dilution, as a positive control. After incubation for 5 days (120 h), 30 μL of 0.5 mM solution of chlorophenol red substrate β-galactopyranoside (CPRG) in PBS, with 0.9% v/v of Igepal CA-630, was added. After incubation for 1.5 h, absorbance was measured at λ = 570 nm using a plate reader.^37^

### Molecular Modeling

The definition of the most stable tautomeric forms and the protonation states of the flavanones (2*S*)-strobopinin and (2*S*)-cryptostrobin at pH = 7.4 was made through MarvinSketch p*K*_a_ plugin (Chemaxon, 2023). The structures were prepared and had their energies minimized with the PM6 semiempirical method in the Spartan’20 program (Wave function, Inc.).^38^

The 2.75 Å resolution crystallographic structure of glycolitic glyceraldehyde-3-phosphate dehydrogenase (EC 1.2.1.12) of *T. cruzi* (TcGAPDH), containing as cocrystallized ligands 3-hydroxy-2-oxo-4-phophonoxy-butyl-phosphonic acid (S70) and NAD^+^, was extracted from the protein data bank (code 1QXS).^34^ Flavanones docking was explored with GOLD2023.2.0 (CCDC) at the enzyme’s orthosteric site located in chain A. Hydrogen atoms were added with the default program method. Seven water molecules involved in the ligand interaction at the orthosteric site were maintained (H_2_O: 867, 874, 880, 924, 945, 973, and 975) during the docking.

The fitness score function was selected through a redocking study in four different environments, considering catalytic residues (Cys166 and His194) in neutral and ionized forms, and the presence and absence of the NAD^+^ cofactor. After extracting the inhibitor, the docking of the flavanones was proceeded with the selected scoring function into a 12 Å binding site radius from Cys166. Flavanones were docked in the high flexibility mode of the GOLD genetic algorithm. The generated poses were analyzed with Discovery Studio (Dassault Systèmes). The best solutions were completely reoptimized with the molecular orbital method PM7 available in Mopac2016 (Stewart Computational Chemistry), from which the interaction enthalpies could be calculated. To simulate the water environment, the COSMO continuum model was used with an appropriate dielectric constant.^39,40^ The enthalpy of interaction between ligand and enzyme (ΔH_int_) was calculated as the difference between the ΔH_f_ of the enzyme-ligand complex and the sum of the values of ΔH_f_ of the isolated enzyme and ligand.^41^

GOLD is able to dock covalently bound inhibitors by specifying which ligand atom is bonded to which protein atom. This docking procedure was also explored by us with the flavanones. The program assumes that there is just one atom linking the ligand to the protein, which was the S atom of the catalytic cysteine residue of TcGAPDH (Cys166). In order to make sure that the geometry of the bound ligand is correct, the angle-bending potential from the Tripos Force Field is incorporated into the fitness function.

Swiss-ADME was used to predict ADME parameters, pharmacokinetic and druglike properties of the flavanones.^42^

## Conclusions

This study analyzed ethanolic extracts of Brazilian geopropolis from Melipona mondury and evaluated its activity against *Trypanosoma cruzi*. The extract was partitioned with hexane and dichloromethane, indicating the presence of active compounds. Two *C*-methylated flavanones, (2*S*)-strobopinin and (2*S*)-cryptostrobin, were isolated from the dichloromethane fraction and identified as novel components of this Brazilian geopropolis. Although both flavanones showed comparable levels of activity, (2*S*)-cryptostrobin proved to be the most selective compound. Molecular modeling studies suggested as a possible mechanism of action for the compounds the inhibition of a parasite’s enzyme, glyceraldehyde-3-phosphate dehydrogenase, in accordance with results from the literature describing similar compounds with the same activity. This study reinforces the potential of geopropolis as a rich source of natural bioactives with important antiparasitic activity.

## Supplementary Material



## Notes

Figure S1: HPLC of the crude extract of geopropolis from M. mondury (in black) and the hexane (in blue), dichloromethane (in pink) and hydroalcoholic (green) fractions. Figure S2: HPLC and UV spectrum of the substance S1. Figure S3: ^1^H NMR spectrum (500 MHz, CDCl_3_) of (2*S*)-strobopinin. Figure S4: ^13^C NMR spectrum (125 MHz, CDCl_3_) of (2S)-strobopinin. Figure S5. ^1^H-^13^C HMBC spectrum of (2*S*)-strobopinin. Figure S6: HPLC and UV spectrum of the substance S2. Figure S7: ^1^H NMR spectrum (500 MHz, CDCl_3_) of (2*S*)-cryptostrobin. Figure S8: ^13^C NMR spectrum (125 MHz, CDCl_3_) of (2*S*)-cryptostrobin. Figure S9: ^1^H-^13^C HMBC spectrum of (2*S*)-cryptostrobin. Figure S10: Mass spectrum of (2*S*)-strobopinin and (2*S*)-cryptostrobin. Figure S11: Fragmentation proposal for isolated natural flavanones (2*S*)-strobopinin and (2*S*)-cryptostrobin. Table S1: Calculated enthalpies of formation (DHf) in kcal·mol^–1^ (PM7 method) for the ligands strobopinin and cryptostrobin and for the site-ligand complexes obtained with glyceraldehyde 3-phosphate dehydrogenase (GAPDH – PDB id: 1QXS) (PDF)
