# Supplementary material for: C‑methylated Flavanones with Antitrypanosomal Activity Isolated of Geopropolis from Melipona mondury
Source: ACS Omega. 2025 May 27;10(22):23397–408. doi: 10.1021/acsomega.5c01804 (PMC12163691; doi:10.1021/acsomega.5c01804)
Supplement: Supplementary file 1 [file ao5c01804_si_001.pdf]

# Supporting Information

**for**

*C*-methylated flavanones with anti-trypanosomal activity isolated of geopropolis from *Melipona mondury*.

Rafael Ferreira dos Santos [a], Afonso Santine M. M. Velez [a], Gabriel Fulgencio dos Santos [a], Paulo Pitasse dos Santos [b], Bruno Sena de Oliveira [a], Marco Edilson Freire de Lima [a], Carlos Mauricio R. Sant'Anna [a], Raimundo Braz Filho [a,c], Debora Decote-Ricardo [d] and Rosane Nora Castro [a*]

[a] Universidade Federal Rural do Rio de Janeiro, Instituto de Química, Departamento de Química Orgânica, CEP 23897-000, Seropédica-RJ, Brasil.

[b] Leicester Institute of Structural and Chemical Biology, University of Leicester, University Road, Leicester, LE1 7RH, UK
School of Chemistry, University of Leicester, University Road, Leicester, LE1 7RH, UK

[c] Universidade Estadual do Norte Fluminense Darcy Ribeiro – UENF, Centro de Ciência e Tecnologia – CCT, Departamento de Química de Produtos Naturais, Laboratório de Ciências Químicas – LCQUI, Campos dos Goytacazes, RJ, Brasil

[d] Universidade Federal Rural do Rio de Janeiro, Instituto de Veterinária, CEP 23897-000, Seropédica-RJ, Brasil.

Corresponding author. Tel.: +55 (21) 99103-1111

*E-mail address*: nora@ufrrj.br

**Figure S1**: HPLC of the crude extract of geopropolis from *M. mondury* and its fractions.

**Figure S2**: HPLC and UV spectrum of the substance S1.

**Figure S3**: $^{1}H$ NMR spectrum (500 MHz, $CDCl_3$) of (2*S*)-strobopinin.

**Figure S4**: $^{13}C$ NMR spectrum (125 MHz, $CDCl_3$) of (2*S*)-strobopinin.

**Figure S5** $^{1}H$ **-**$^{13}C$ HMBC spectrum of (2*S*)-strobopinin.

**Figure S6**: HPLC and UV spectrum of the substance S2.

**Figure S7**: $^{1}H$ NMR spectrum (500 MHz, $CDCl_3$) of (2*S*)-cryptostrobin.

**Figure S8**: $^{13}C$ NMR spectrum (125 MHz, $CDCl_3$) of (2*S*)-cryptostrobin.

**Figure S9**: $^{1}H$ -$^{13}C$ HMBC spectrum of (2S)-cryptostrobin.

**Figure S10**: Mass spectrum of (2*S*)-strobopinin and (2*S*)-cryptostrobin

**Figure S11**: Fragmentation proposal for isolated natural flavanones (2*S*)-strobopinin and (2*S*)-cryptostrobin.

**Table S1:** Calculated enthalpies of formation ($DH_f$) in kcal.mol$^{-1}$ (PM7 method) for the ligands strobopinin and cryptostrobin and for the site-ligand complexes obtained with glyceraldehyde 3-phosphate dehydrogenase (GAPDH – PDB id: 1QXS)

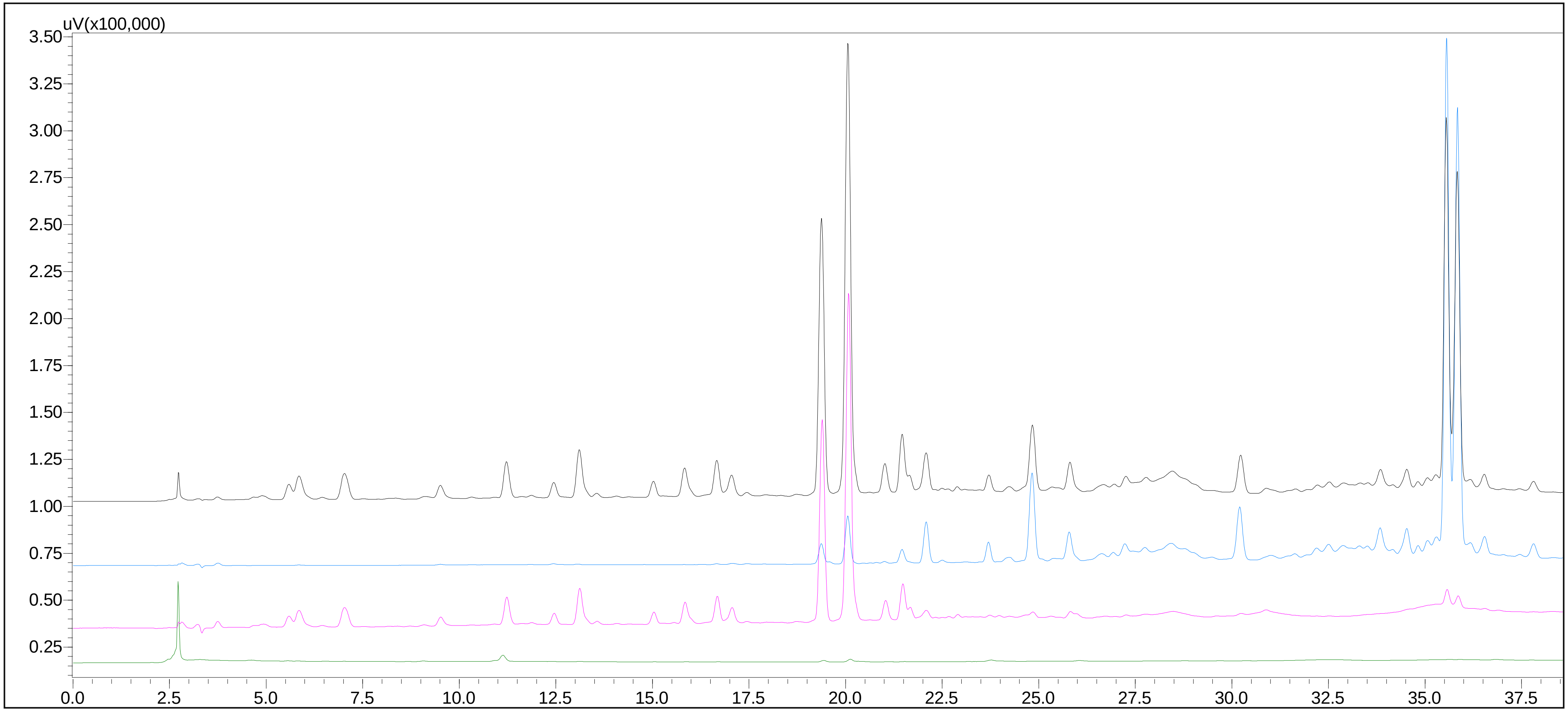


**Figure S1**: HPLC of the crude extract of geopropolis from *M. mondury* (in black) and the hexane (in blue), dichloromethane (in pink) and hydroalcoholic (green) fractions.

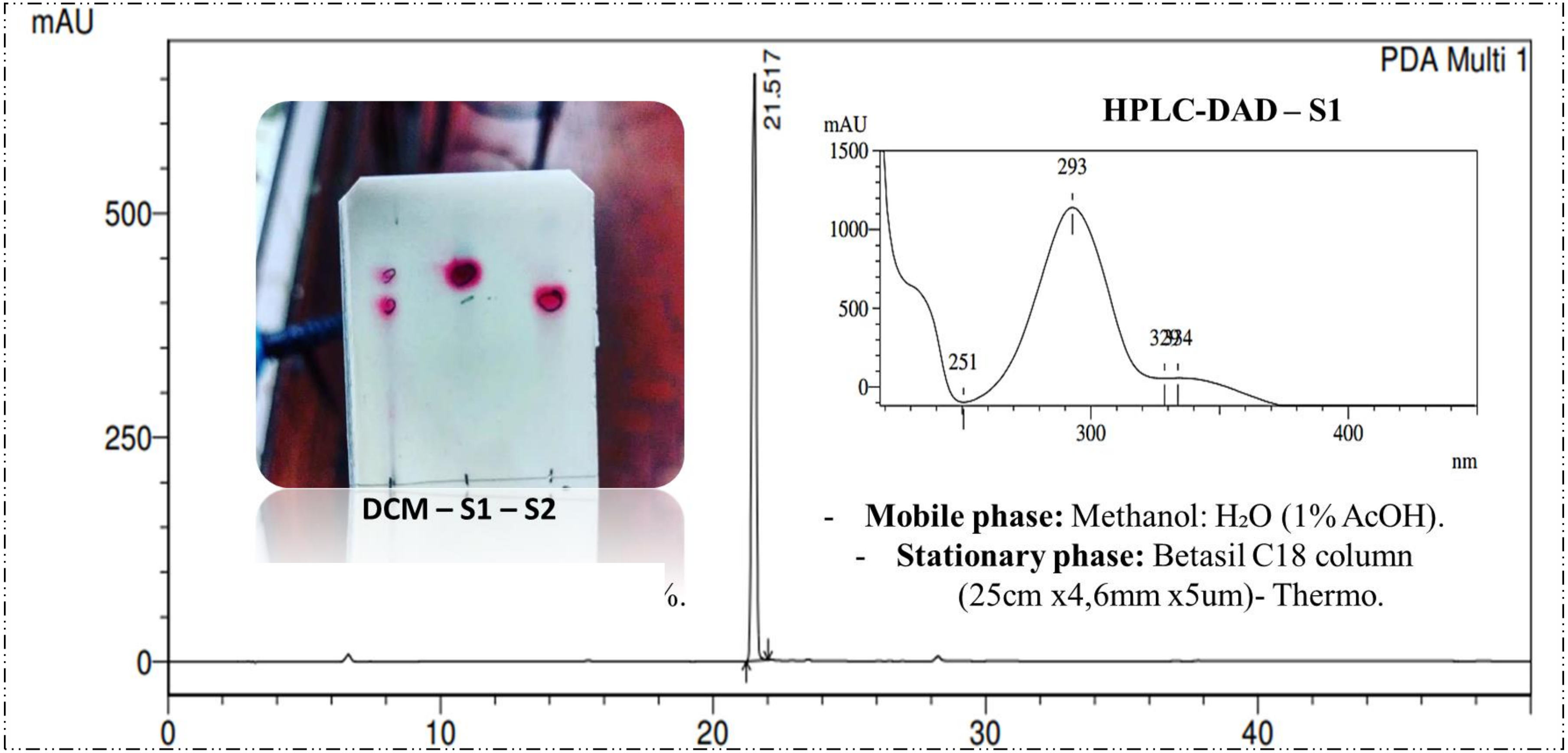


**Figure S2:** HPLC and UV spectrum of the substance S1.

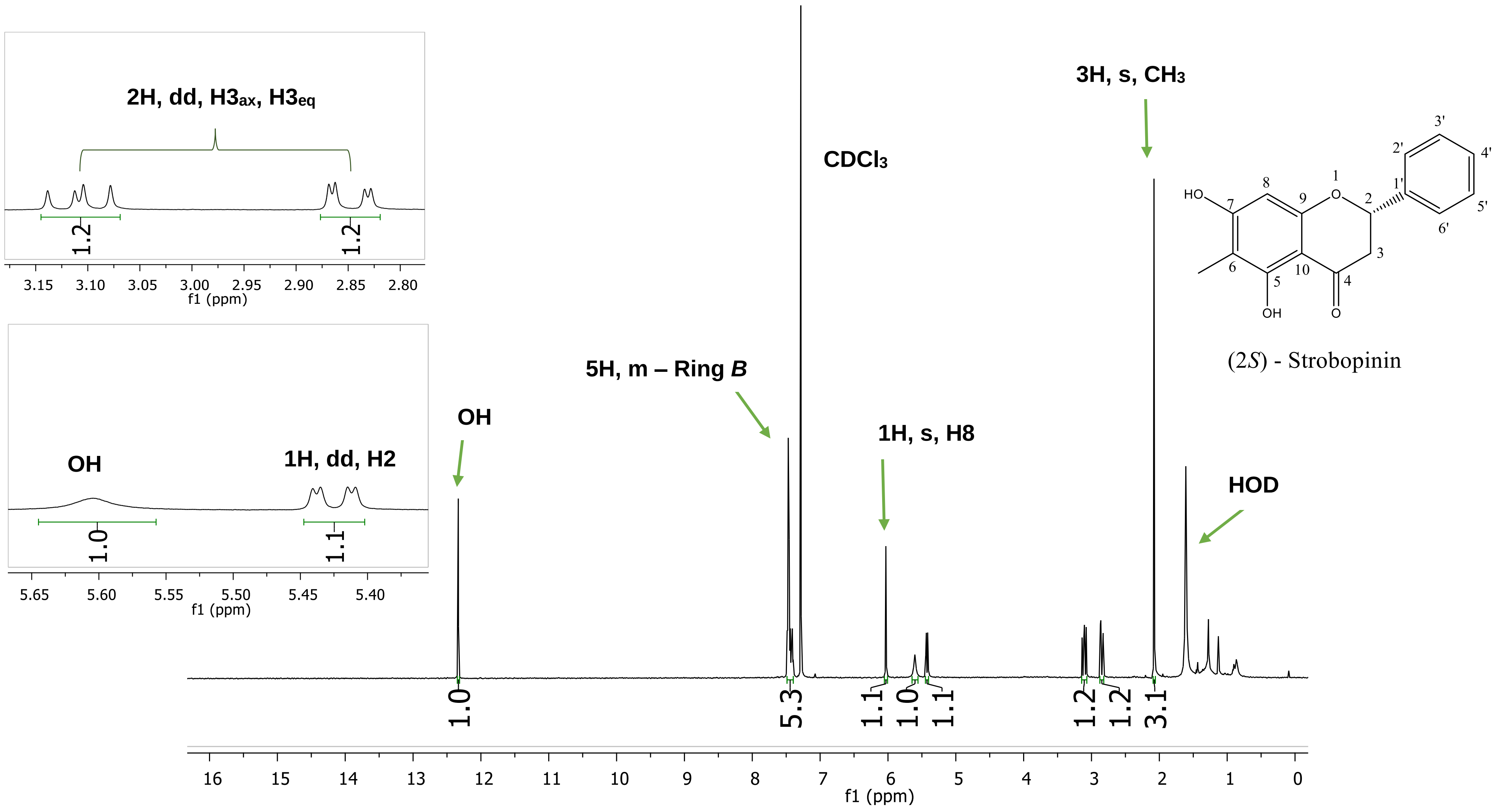
2H, dd, H3ax, H3eq
1.2
1.2
3.15 3.10 3.05 3.00 2.95 2.90 2.85 2.80
f1 (ppm)
OH
1H, dd, H2
1.0
1.1
5.65 5.60 5.55 5.50 5.45 5.40
f1 (ppm)
CDCl3
3H, s, CH3
5H, m – Ring B
OH
1H, s, H8
HOD
HO
OH
O
(2S) - Strobopinin
1.0
5.3
1.1
1.0
1.1
1.2
1.2
3.1
16 15 14 13 12 11 10 9 8 7 6 5 4 3 2 1 0
f1 (ppm)

**Figure S3**: $^{1}$H NMR spectrum (500 MHz, $CDCl_3$) of (2*S*)-strobopinin.

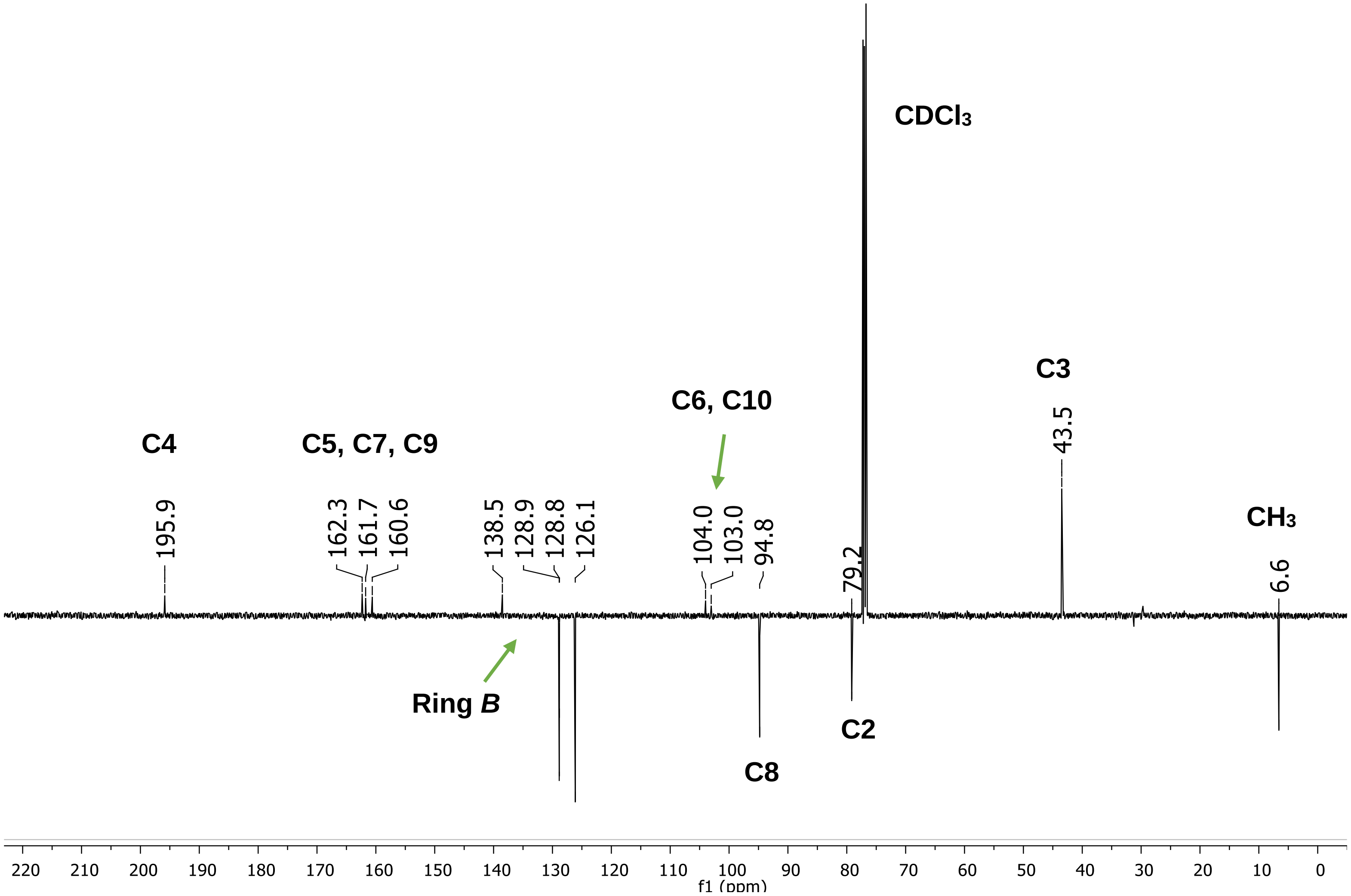

**Figure S4**: $^{13}C$ NMR spectrum (125 MHz, $CDCl_3$) of (2*S*)-strobopinin.

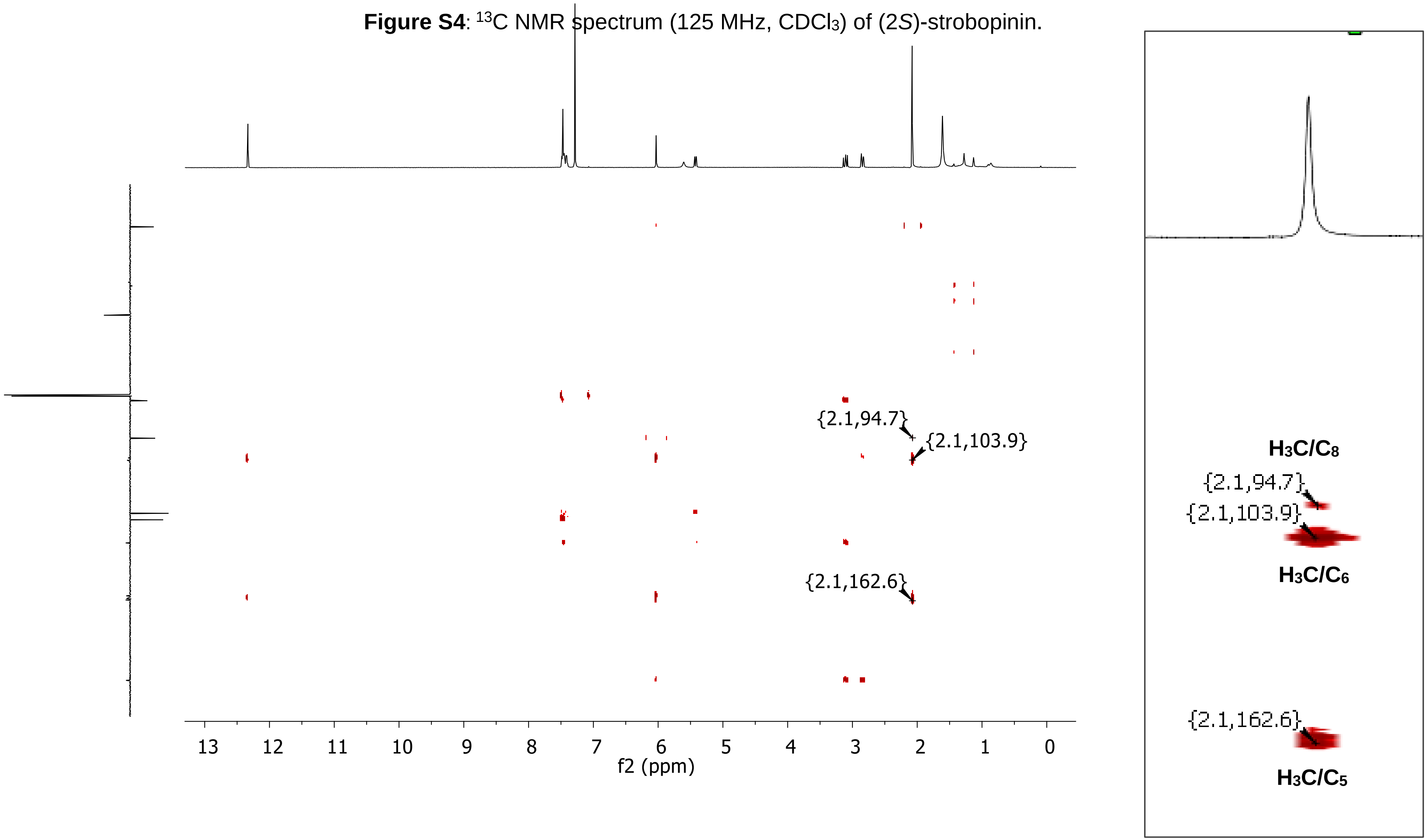

**Figure S5**: $^{1}H$ -$^{13}C$ HMBC spectrum of (2*S*)-strobopinin.

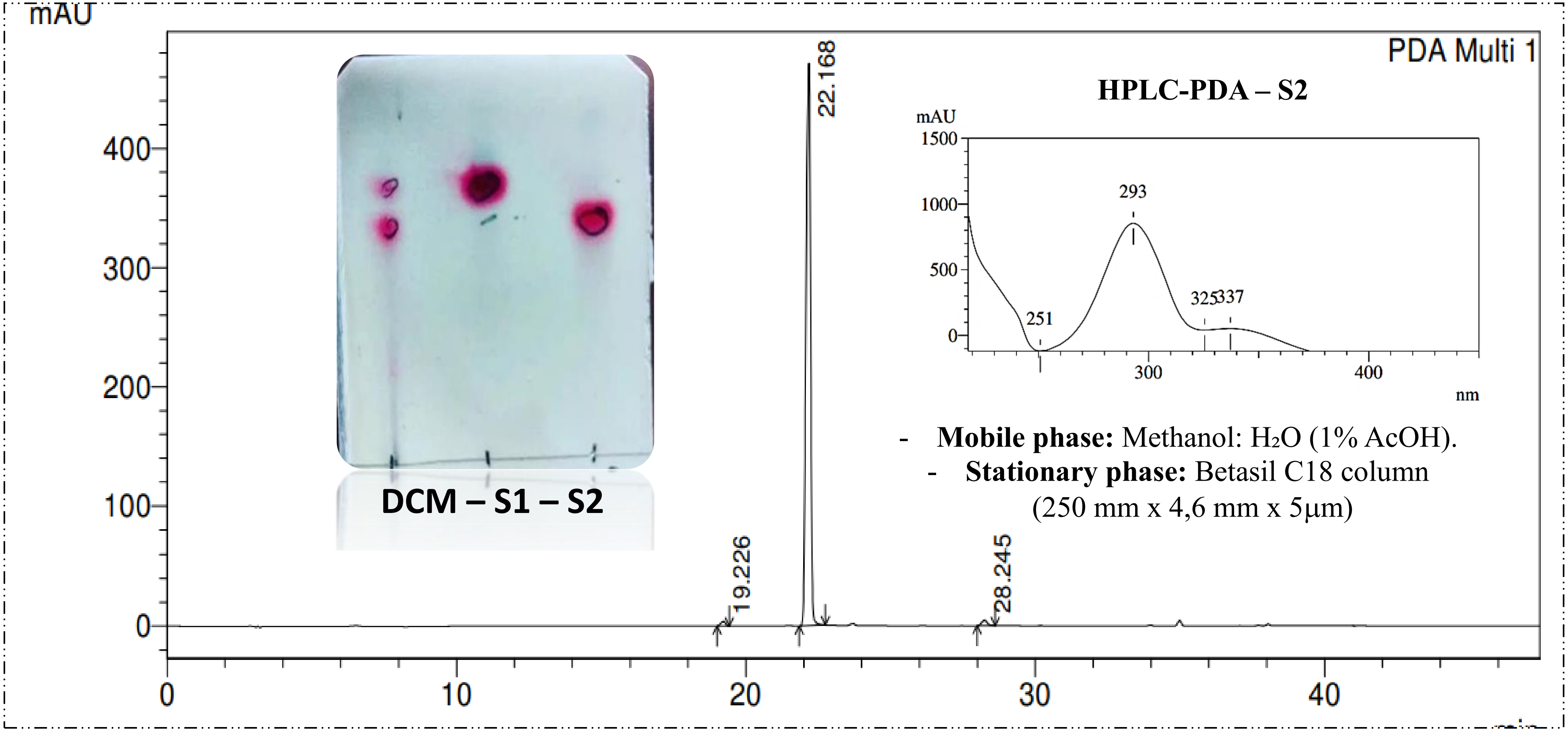


**Figure S6:** HPLC and UV spectrum of the substance S2.

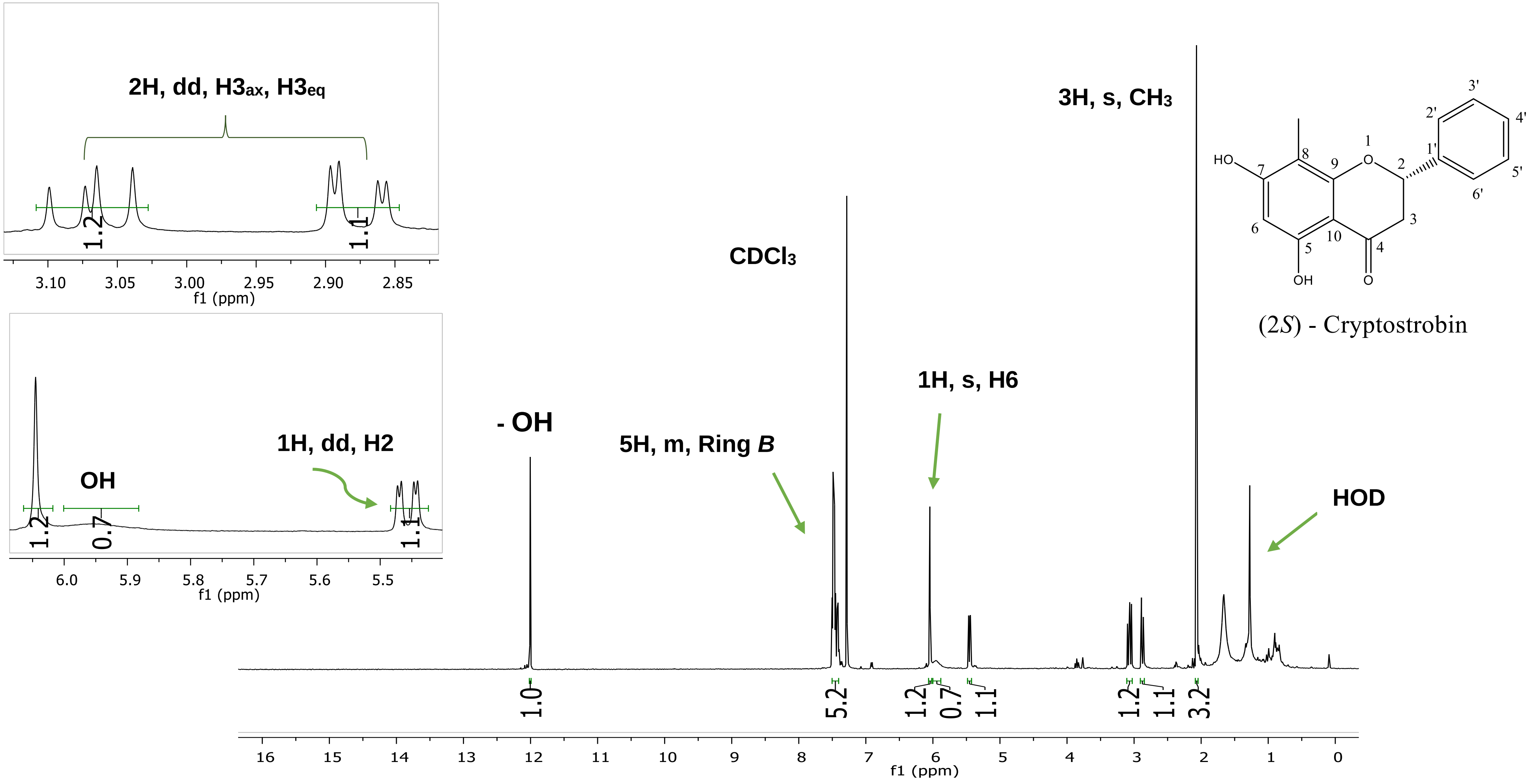

2H, dd, H3ax, H3eq
1.2
1.1
3.10
3.05
3.00
2.95
2.90
2.85
f1 (ppm)
OH
1H, dd, H2
1.2
0.7
1.1
6.0
5.9
5.8
5.7
5.6
5.5
f1 (ppm)
- OH
5H, m, Ring *B*
CDCl3
1H, s, H6
3H, s, CH3
HOD
1.0
5.2
1.2
0.7
1.1
1.2
1.1
3.2
16
15
14
13
12
11
10
9
8
7
6
5
4
3
2
1
0
f1 (ppm)
HO
OH
O
(2*S*) - Cryptostrobin

**Figure S7**: $^{1}$H NMR spectrum (500 MHz, $CDCl_3$) of (2*S*)-cryptostrobin

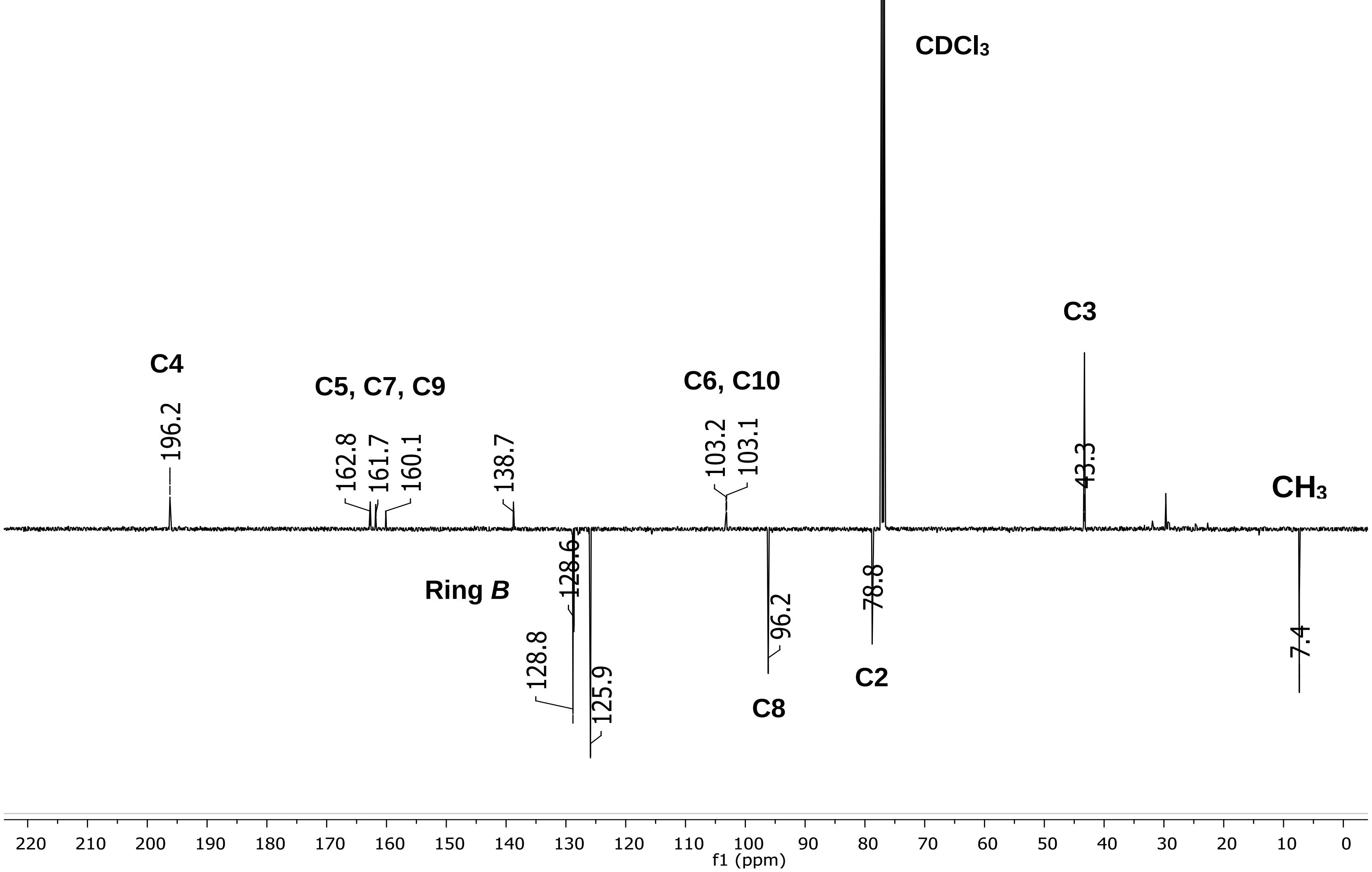

**Figure S8**: $^{13}$C NMR spectrum (125 MHz, $CDCl_3$) of (2*S*)-cryptostrobin

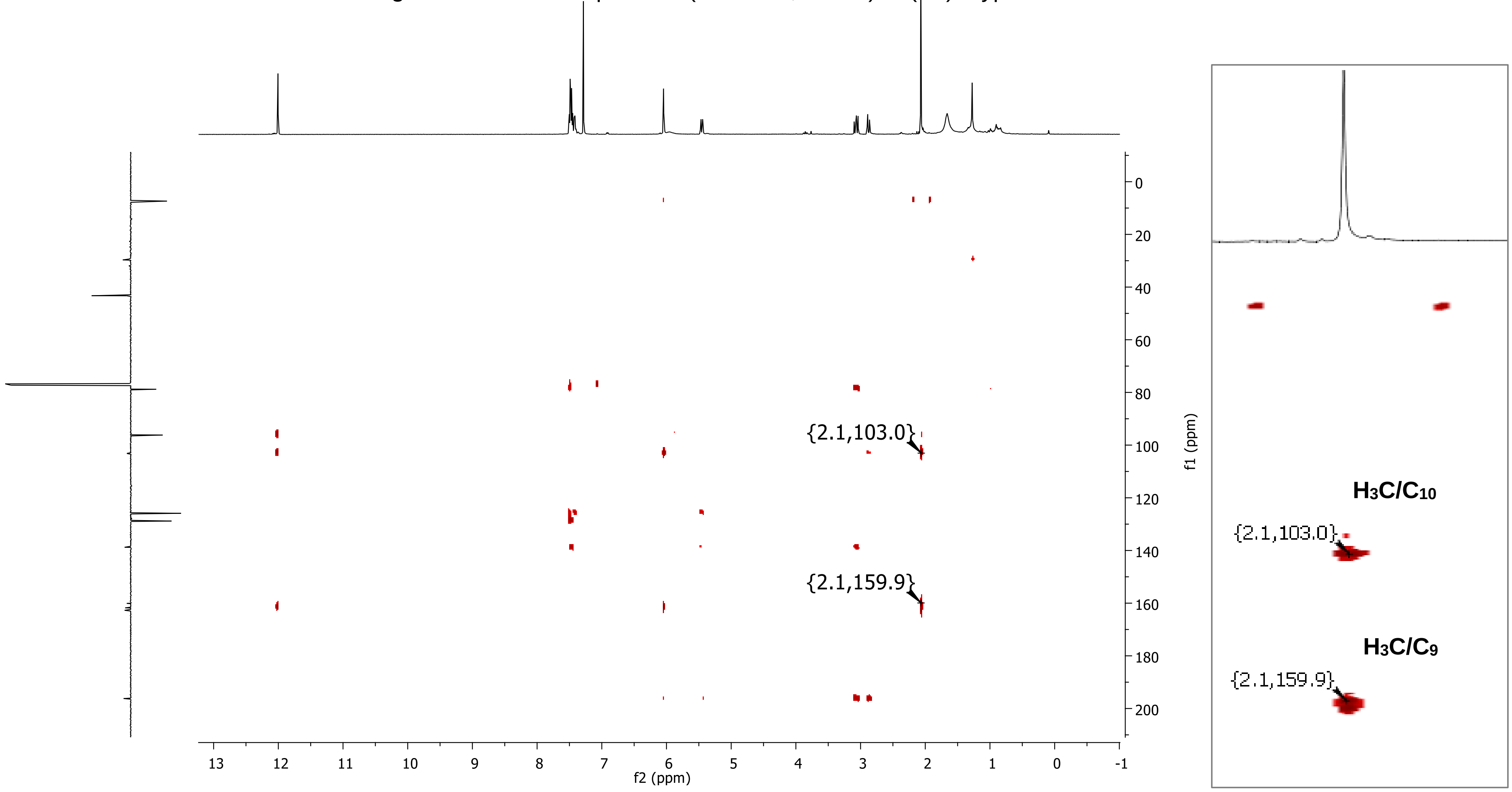

**Figure S9**: $^{1}$H -$^{13}$C HMBC spectrum of (2*S*)-cryptostrobin.

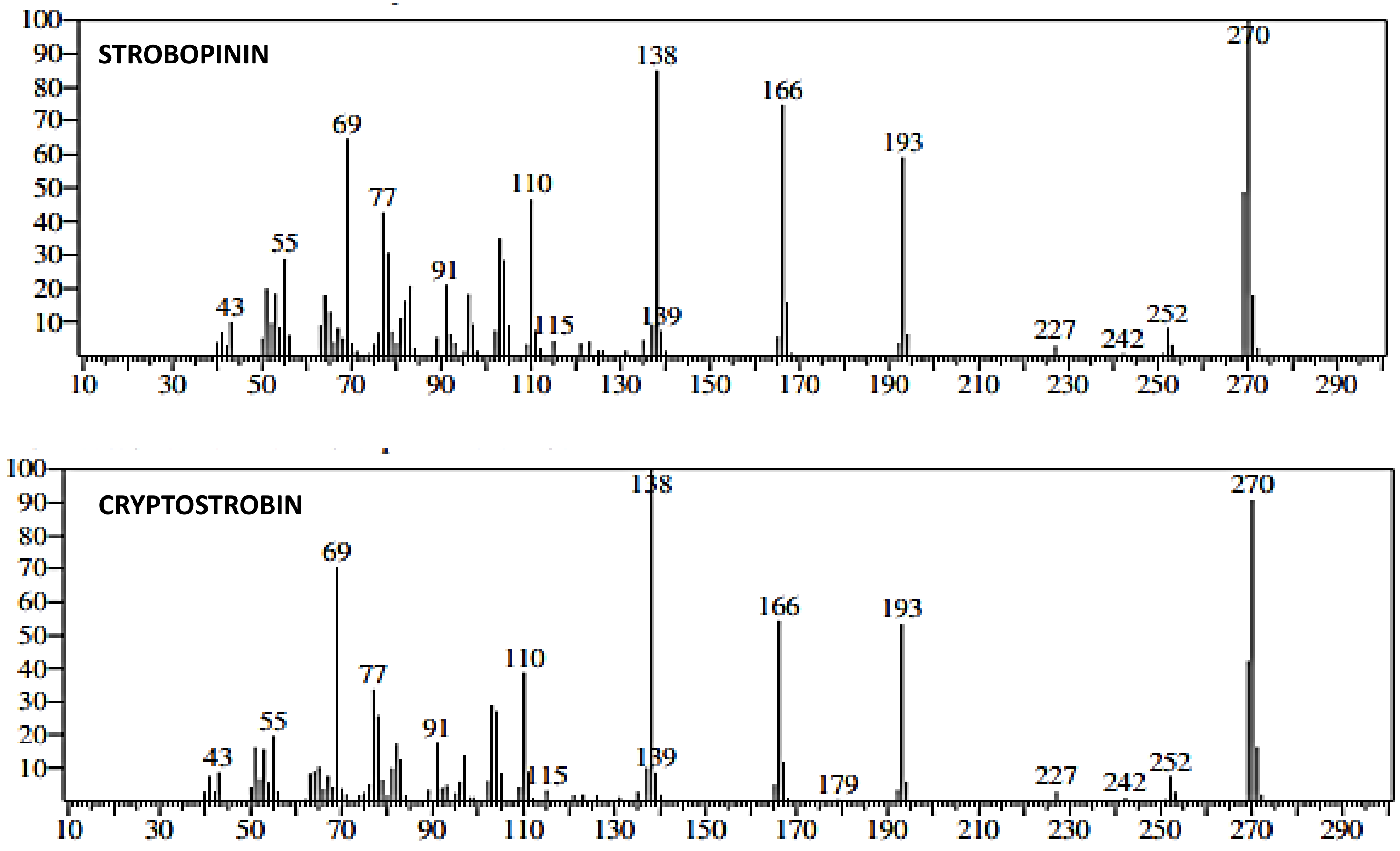


**Figure S10:** Mass spectra of (2*S*)-strobopinin and (2*S*)-cryptostrobin

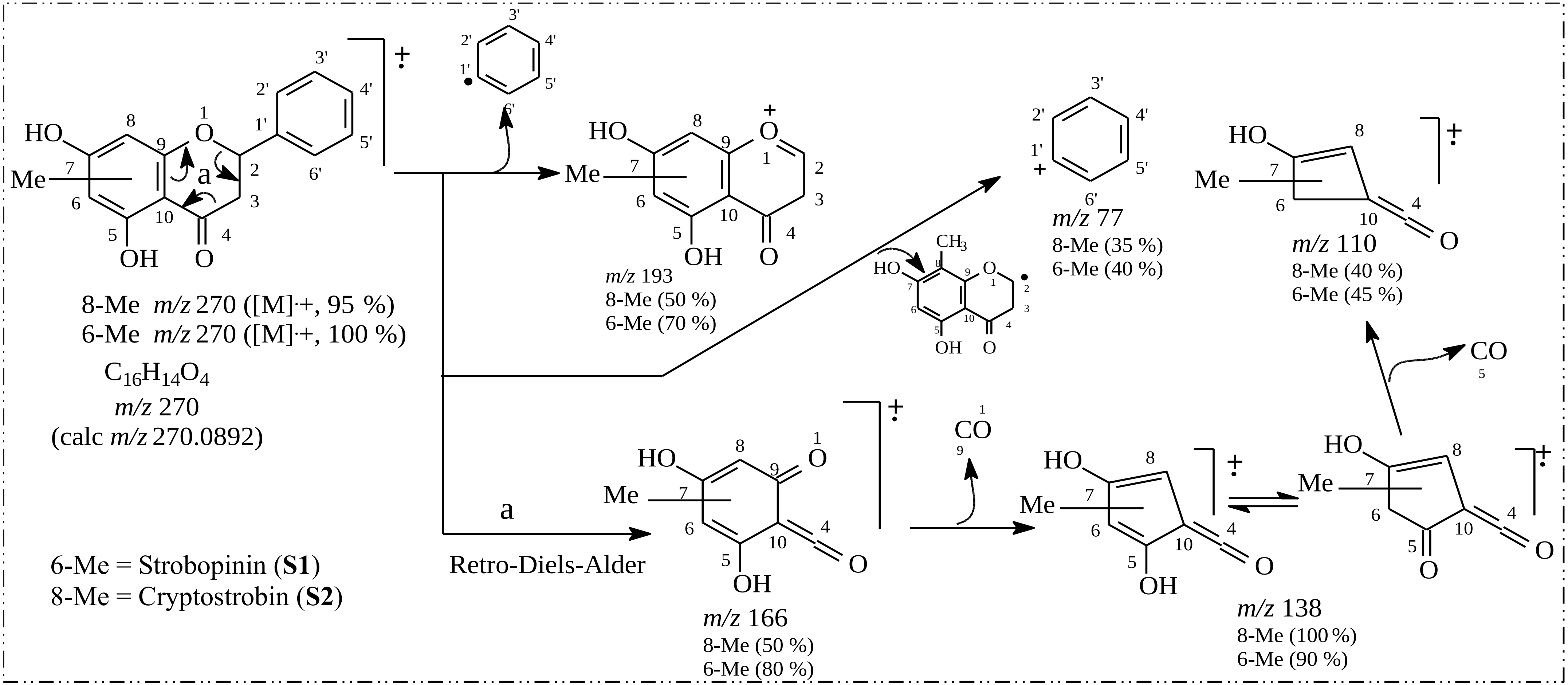
8-Me m/z 270 ([M]·+, 95 %)
6-Me m/z 270 ([M]·+, 100 %)
C16H14O4
m/z 270
(calc m/z 270.0892)
6-Me = Strobopinin (S1)
8-Me = Cryptostrobin (S2)
Retro-Diels-Alder
m/z 193
8-Me (50 %)
6-Me (70 %)
m/z 77
8-Me (35 %)
6-Me (40 %)
m/z 110
8-Me (40 %)
6-Me (45 %)
m/z 166
8-Me (50 %)
6-Me (80 %)
m/z 138
8-Me (100 %)
6-Me (90 %)
CO
CO

**Figure S11:** Fragmentation proposal for isolated natural flavanones (2*S*)-strobopinin and (2*S*)-cryptostrobin.

**Table S1:** Calculated enthalpies of formation ($\Delta H_f$) in kcal.mol$^{-1}$ (PM7 method) for the ligands strobinin and cryptostrobin and for the site-ligand complexes obtained with glyceraldehyde 3-phosphate dehydrogenase (GAPDH – PDB id: 1QXS)

| **Ligand** | $\Delta H_f^{complex}$ | $\Delta H_f^{ligand}$ | $\Delta H_{int}^{1QXS}$ |
|---|---|---|---|
| Strobopinin | -32486.54 | -147.99 | -307.76 |
| Cryptostrobin | -32492.32 | -143.93 | -317.60 |

The interaction enthalpy ($\Delta H_{int}$) was calculated for each compound according to the equation:

$$\Delta H_{int} = \Delta H_f^{complex} - \left(\Delta H_f^{site} + \Delta H_f^{ligand}\right)$$

where $\Delta H_f^{site}$ = -32030.79 kcal.mol$^{-1}$
